# The tetraspanins CD151 and Tspan8 are essential exosome components for the crosstalk between cancer initiating cells and their surrounding

**DOI:** 10.18632/oncotarget.2958

**Published:** 2014-12-10

**Authors:** Shijing Yue, Wei Mu, Ulrike Erb, Margot Zöller

**Affiliations:** ^1^ Department of Tumor Cell Biology, University Hospital of Surgery, Heidelberg, Germany

**Keywords:** Tetraspanins, exosomes, metastasis, matrix degradation, EMT

## Abstract

Tspan8 and CD151 are metastasis-promoting tetraspanins and a knockdown (kd) of Tspan8 or CD151 and most pronounced of both tetraspanins affects the metastatic potential of the rat pancreatic adenocarcinoma line ASML. Approaching to elaborate the underlying mechanism, we compared ASML^wt^, -CD151^kd^ and/or Tspan8^kd^ clones. We focused on tumor exosomes, as exosomes play a major role in tumor progression and tetraspanins are suggested to be engaged in exosome targeting.

ASML-CD151/Tspan8^kd^ cells poorly metastasize, but regain metastatic capacity, when rats are pretreated with ASML^wt^, but not ASML-CD151^kd^ and/or -Tspan8^kd^ exosomes. Both exosomal CD151 and Tspan8 contribute to host matrix remodelling due to exosomal tetraspanin-integrin and tetraspanin-protease associations. ASML^wt^ exosomes also support stroma cell activation with upregulation of cytokines, cytokine receptors and proteases and promote inflammatory cytokine expression in hematopoietic cells. Finally, CD151-/Tspan8-competent exosomes support EMT gene expression in poorly-metastatic ASML-CD151/Tspan8^kd^ cells. These effects are not seen or are weakened using ASML-CD151^kd^ or -Tspan8^kd^ exosomes, which is at least partly due to reduced binding/uptake of CD151- and/or Tspan8-deficient exosomes.

Thus, CD151- and Tspan8-competent tumor exosomes support matrix degradation, reprogram stroma and hematopoietic cells and drive non-metastatic ASML-CD151/Tspan8^kd^ cells towards a motile phenotype.

## INTRODUCTION

Tumors are a heterogeneous mixture of cells, where a small population of so called cancer-initiating cells (CIC) [1,2] is supposed to account for tumor progression including epithelial-mesenchymal transition (EMT) and metastatic settlement and growth [3,4]. With the description of the phenomenon of a premetastatic niche [5] the question arose on the factors that modulate selected distant organs to facilitate disseminated tumor cell embedding [6]. Similar, in concern on the shift towards EMT, the question remains to be answered, why preferentially cells at the tumor rim become engaged and which trigger they receive [7,8]. Particularly for the establishment of a premetastatic niche strong evidence is accumulating that exosomes are the major actors [9-11].

Exosomes, 40-100nm membrane vesicles of endocytic origin [12], are secreted by many cell types, and abundantly by tumor cells [13]. Exosomes are suggested to be the most important intercellular communicators [14,15]. They are defined by size, buoyant density, lipid composition, and protein markers [16]. Their homogeneous size is a major criterion to differentiate from size-variable apoptotic blebs, microparticles and microvesicles [17]. Exosomes are also characterized by a panel of constitutive markers [18], which relate to their origin from endocytosis / early endosomes and include, beside others, components of endocytosis prone membrane domains and of the fission, scission and vesicular transport machineries [19]. One type of endocytosis prone membrane microdomains, tetraspanin-enriched microdomains (TEM) [20], apparently play a major role, as tetraspanins are most widely used to characterize extracellular vesicles as exosomes [21,22]. Furthermore, the net which tetraspanins form between themselves and a multitude of associating molecules is maintained during internalization and intracellular traffic such that tetraspanin webs are recovered in exosomes [23,24]. This feature becomes of particular interest for tetraspanins that are engaged in metastasis formation, which is well known for CD151 and Tspan8 [25,26], the latter also being a CIC marker in pancreatic adenocarcinoma [27]. It is essential to mention that exosomes also contain mRNA and miRNA [16,28] and that all components of exosomes, lipids, proteins, mRNA and miRNA are function competent [29]. Furthermore, exosomes are found in all body fluids [30,31] and can bind to or be taken up by selected target cells, where exosomal tetraspanin webs are involved in target cell selection [32].

Tetraspanins, a family of 4-transmembrane proteins [33], act as “molecular facilitators” modulating, stabilizing or preventing activities of associated molecules [34]. They promote spreading, migration and cable formation by adjusting integrin compartmentalization, internalization, recycling and signaling [35]. By modulating biosynthesis and activation of associated molecules, like MMPs, they influence invasiveness [36]. Though these activities are generally fitting a contribution to tumor progression, only CD151 and Tspan8 were described as metastasis-promoting in several tumor systems, with no opposing findings reported so far [25,26,37-40]. CD151 preferentially associates with laminin (LN)-binding integrins [41,42], which supports migration via integrin trafficking and activation by Ras, Rac1 and Cdc42 recruitment [43-45]. CD151 also regulates cell motility via proteases [46]. CD151 induces MMP9 expression via CD151-associated integrin signaling [47] and anchors MMP7 at the cell membrane by MMP9 binding [48]. CD151 also associates with MMP14 [49] and regulates ADAM10 and ADAM17 activity by recruitment into TEM [46]. Finally, a cathepsinB or uPAR knockdown inhibits CD151-α3β1-mediated cell adhesion and invasion [50]. Finally, a CD151 molecule with a mutation of the sorting motif in the C-terminal domain markedly attenuates endocytosis of CD151-associated α3β1, α5β1 and α6β1 [36,43], which could affect CD151-supported exosome activity.

We recently uncovered by a CD151 knockdown (CD151^kd^) in a rat pancreatic adenocarcinoma that CD151 promotes adhesion, which is overcome by CD151-associated MMPs degrading the extracellular matrix [51]. Tspan8 by associating with α6β4 [52,53] contributes to paxillin and FAK activation and supports motility [51]. Accordingly, both ASML-CD151^kd^ and ASML-Tspan8^kd^ cells displayed reduced metastatic capacity, the deficits suggesting supplementary activities of the two tetraspanins. To substantiate this assumption, we generated double ASML-CD151/Tspan8^kd^ clones, which poorly metastasize. Based on this finding, the known contribution of exosomes to the metastatic process [9-11] and the functional importance of exosomal tetraspanins [25], we controlled for the contribution of exosomal CD151 and Tspan8 in tumor progression. ASML-CD151/Tspan8^kd^ exosomes do not support metastatic settlement, they have severe defects in extracellular matrix (ECM) degradation, poorly stimulate chemokine and chemokine receptor expression and are not generating an inflammatory milieu. Finally, only ASML^wt^ exosomes support EMT in poorly metastatic ASML-CD151/Tspan8^kd^ cells.

## RESULTS

We recently described that CD151 and Tspan8 distinctly, but supplementarily support metastasis, where Tspan8 predominantly accounts for motility and CD151 for adhesion and matrix degradation [51]. We here evaluate the contribution of exosomal tetraspanins to tumor progression, using as additional control an ASML-CD151/Tspan8^kd^ line.

### A CD151 / Tspan8 knockdown is accompanied by loss in metastatic tumor growth

**Figure 1:**
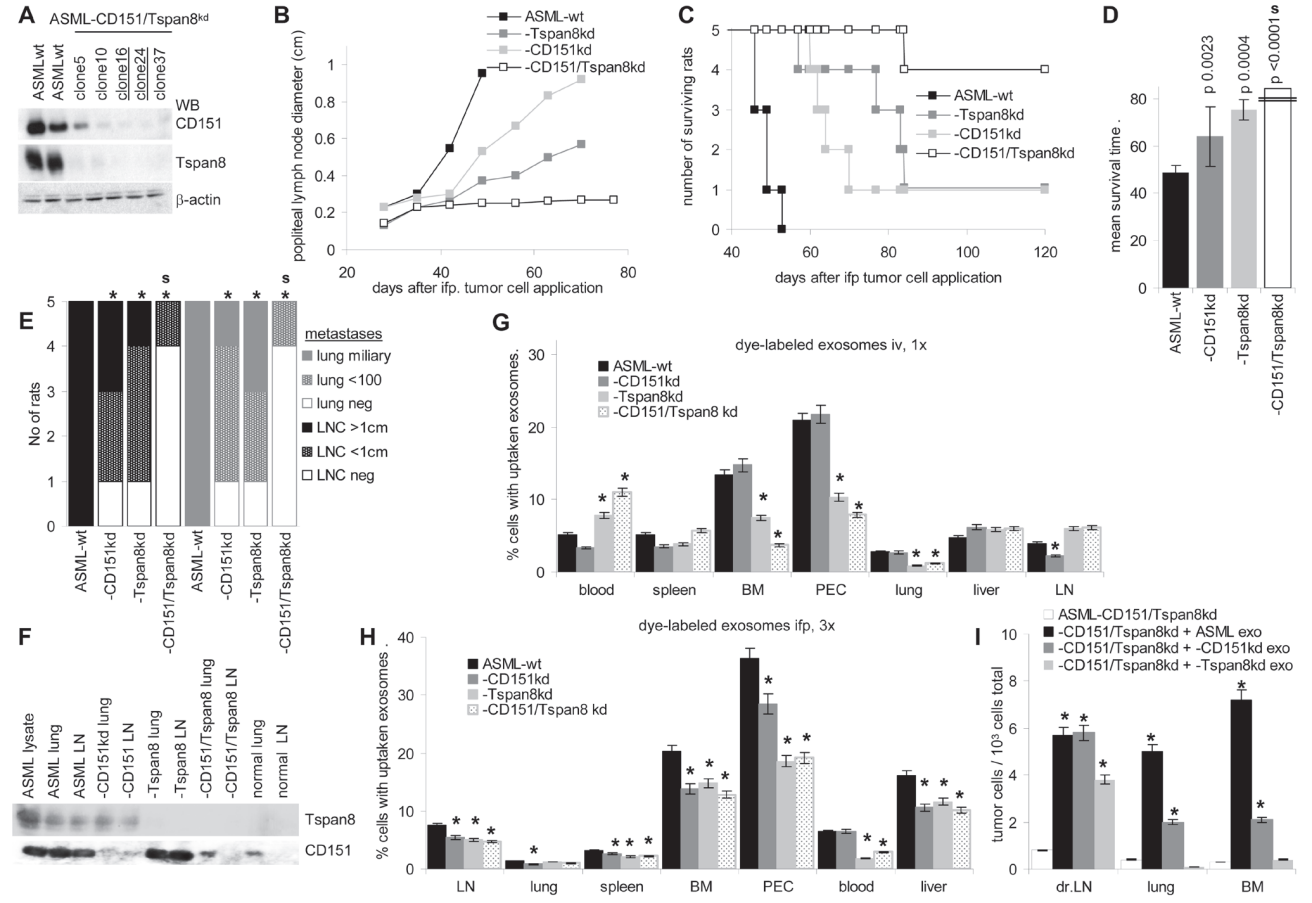
CD151 and Tspan8 requirement for metastasis formation and for exosome distribution Clones 16 and 24 were used for further experiments. (B-F) BDX rats (5/group) received an ifp injection of 1×10^6^ ASML^wt^ or -CD151^kd^ and/or -Tspan8^kd^ cells. (B) Tumor growth in the popliteal node; (C) survival time and survival rate; (D) mean survival time, indicating significant differences compared to ASML^wt^-bearing rats and between ASML-CD151/Tspan8^kd^ versus -CD151^kd^ or Tpsan8^kd^ bearing rats: s; (E) No of rats with small or large LN metastasis and of rats with no, few or >1000 lung metastases; significant differences compared to ASML^wt^-bearing rats: *, and between ASML-CD151/Tspan8^kd^ versus -CD151^kd^ or Tpsan8^kd^ bearing rats: s; (F) recovery of CD151 and Tspan8 in lung and LN lysates of control and tumor-bearing rats. (G) Rats (3/group) received a single injected of dye-labeled exosomes, iv. Rats were sacrificed after 48h; (H) rats (3/group) received three injected of dye-labeled exosomes in 3d intervals, ifp, and were sacrificed 48h after the last injection; (G,H) lymphatic organs were excised and the recovery of dye-labeled cells (exosome uptake) was evaluated by flow cytometry. The mean±SD of dye-labeled cells is shown; significant differences to the uptake of ASML^wt^ exosomes: *; (I) Rats (5/group) received 1×10^6^ ASML-CD151/Tspan8^kd^ cells ifp and starting at day -6 in 3d intervals, 100μg exosomes, ifp. Rats were scarified after 21d. Recovery of tumor cells in draining LN, lung and BM was evaluated by flow cytometry after staining for the ASML marker C4.4A; the mean No±SD of tumor cells / 10^3^ cells is shown; significant differences to ASML-CD151/Tspan8^kd^ bearing rats: *. A CD151^kd^ or a Tspan8^kd^ retards tumor growth. ASML-CD151/Tspan8^kd^ cells rarely metastasize. ASML-CD151^kd^ and/or ASML-Tspan8^kd^ exosomes are poorly recovered in lymphoid organs, which is accompanied by ASML-Tspan8^kd^ exosome retention at the injection site.

ASML-Tspan8^kd^ cells were transfected with CD151 shRNA, selecting clones 16 and 24 for further studies (Figure 1A). After intrafootpad (ifp) application, the double knockdown clones hardly grew in draining nodes and only 1 of 5 rats developed lung metastasis. As described [51], the survival time of rats receiving ASML-CD151^kd^ or ASML-Tspan8^kd^ cells was prolonged and the tumor burden in LN and lung was reduced compared to ASML^wt^-bearing rats, but did not differ between ASML-CD151^kd^- versus ASML-Tspan8^kd^-bearing rats. Instead, the survival time and the metastatic burden of ASML-CD151/Tspan8^kd^ bearing rats differed significantly from that of ASML-CD151^kd^ or -Tspan8^kd^ bearing animals (Fig.1B-1E). Lymph nodes (LN) and lung of the one rat developing lung metastasis after receiving ASML-CD151/Tspan8^kd^ cells displayed weak CD151 expression that, however, did not exceed the level of normal lung tissue (Fig.1F). Immunohistology staining for CD44v6, which is highly expressed in ASML cells, confirmed the stability of the Tspan8^kd^ and/or CD151^kd^ *in vivo* (Suppl.Fig.1).

**Figure 2:**
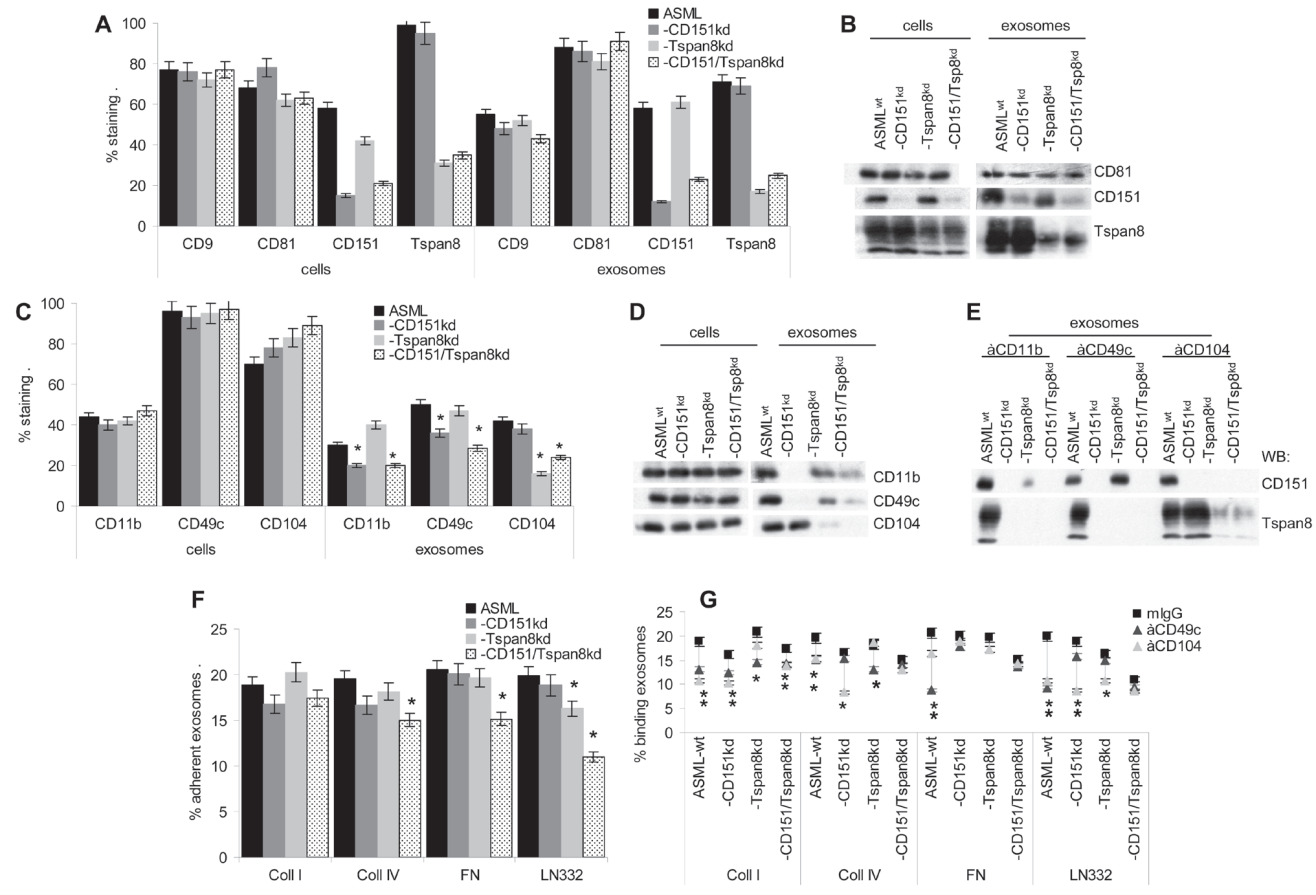
The impact of CD151 and Tspan8 on tetraspanin and adhesion molecule expression in ASML cells and exosomes (A-D) ASML cells and exosomes were stained for expression of the indicated tetraspanins and adhesion molecules; (A,C) Mean±SD (3 assays) of the % stained cells and exosomes; significant differences to ASML^wt^ cells and exosomes: * and (B,D) WB. (E) Exosome lysates of ASML^wt^ and -CD151^kd^ and/or Tspan8^kd^ were precipitated with the indicated anti-integrin antibodies and were blotted with anti-CD151 and anti-Tspan8. (F,G) Dye-labeled exosomes were seeded on matrix-coated plates. Where indicated exosomes were preincubated with antibodies. After 2h incubation, plates were washed and remaining fluorescence was evaluated in an ELISA reader. The mean percent±SD (triplicates) of bound exosomes is shown, (F) differences to ASML^wt^ exosomes: *; (G) differences by antibody preincubation: *. Expression of additional tetraspanins and of integrins is not affected in ASML-CD151^kd^ and/or -Tspan8^kd^ cells. Instead, integrin expression in exosomes is reduced corresponding to the associating tetraspanin. Exosomal CD151 and Tspan8 slightly affect exosome adhesion to matrix proteins.

*In vitro* analysis of ASML-CD151/Tspan8^kd^ cells as compared to -Tspan8^kd^ or -CD151^kd^ cells showed significantly decreased cloning efficacy (Suppl.Fig.2A). *In vitro* wound healing (data not shown) and videomicroscopy revealed unaltered motility of ASML-CD151/Tspan8^kd^ cells compared to that of ASML^wt^ cells, i.e. the opposing activities of CD151 (inhibiting) and Tspan8 (promoting) were waved (Suppl.Fig.2B). The reduced capacity of ASML-CD151^kd^ and ASML-Tspan8^kd^ cells to invade matrigel is further impaired in ASML-CD151/Tspan8^kd^ cells, which completely lost invasiveness (Suppl.Fig.2C). Finally, ASML-Tspan8^kd^ and ASML-CD151/Tspan8^kd^ cells poorly transmigrate through an endothelial monolayer (Suppl.Fig.2D).

Taken together, the major contribution of cellular CD151 and Tspan8 to lymphatic metastasis formation relies on promoting motility (Tspan8) and invasiveness (CD151 and Tspan8), such that ASML-CD151/Tspan8^kd^ cells hardly metastasize. As metastasis formation requires a crosstalk with the host [4], which is suggested to be initiated via exosomes [14,15], we proceeded controlling activities of ASML-CD151^kd^, ASML-Tspan8^kd^ and ASML-CD151/Tspan8^kd^ versus ASML^wt^ exosomes.

### Exosomal CD151 and Tspan8 support metastatic settlement

**Figure 3:**
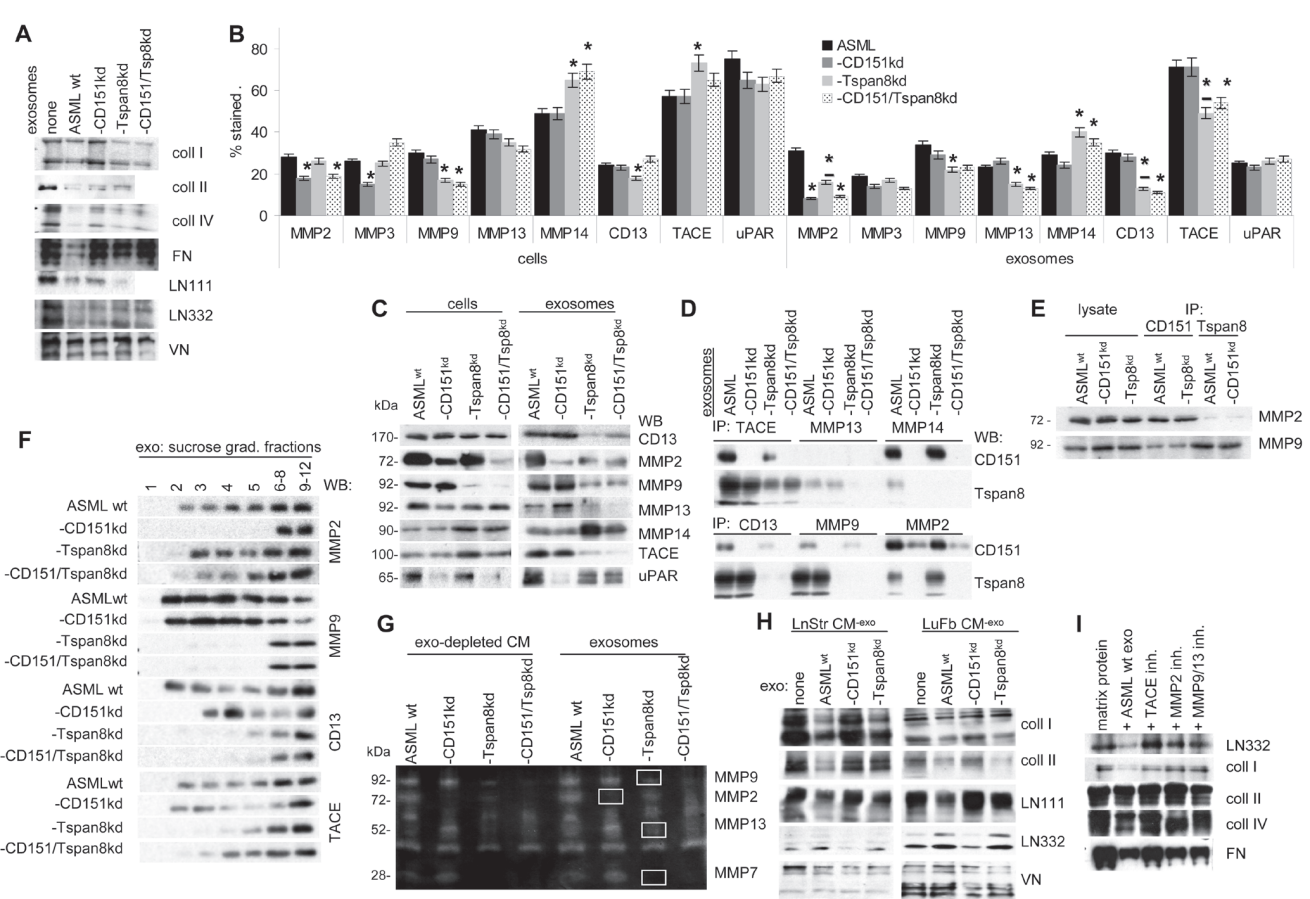
The association of exosomal CD151 and Tspan8 with proteases and the impact on matrix degradation and host cell invasiveness (A) Matrix protein degradation (WB) by ASML^wt^, -CD151^kd^ and/or Tspan8^kd^ exosomes; (B) Protease recovery in ASML^wt^, -CD151^kd^ and/or Tspan8^kd^ cells and exosomes; mean±SD (3 assays) of the percent stained cells and exosome-coated beads; significant differences to ASML^wt^ cells/exosomes: *; significant differences between cells and exosomes: *; (C) protease recovery in ASML^wt^ and -CD151^kd^ and/or -Tspan8^kd^ cells and exosomes as revealed by WB; (D) Coimmunoprecipitation of exosomal CD151 and Tspan8 with proteases and (E) of MMP2 and MMP9 with CD151 and Tspan8; (F) recovery of proteases in light and heavy sucrose density fractions in ASML^wt^, -CD151^kd^ and/or -Tspan8^kd^ exosomes; (G) gelatin (zymography) degradation by ASML^wt^, -CD151^kd^ and/or Tspan8^kd^ exosomes and exosome-depleted conditioned medium; (H) native LnStr and LuFb matrix degradation (WB) by ASML^wt^, -CD151^kd^ and/or Tspan8^kd^ exosomes and (I) inhibition of exosome-mediated matrix protein degradation by TACE, MMP2 and MMP9/MMP13 inhibitors (WB). Recovery of proteases in exosomes is mostly dictated by the association with CD151 and/or Tspan8 such that in the absence of CD151 mostly MMP2 and in the absence of Tspan8 mostly MMP9 expression / activity are strongly reduced. Exosomal TACE activity apparently depends on both Tspan8 and CD151. Reduced exosomal protease recovery has consequences on matrix protein, matrigel and native matrix degradation.

ASML^wt^ exosomes are recovered in all lymphoid organs 48h after intravenous (iv) application. Recovery of ASML-CD151^kd^ exosomes is only reduced in LN. Recovery of ASML-Tspan8^kd^ and -CD151/Tspan8^kd^ exosomes is reduced in bone marrow (BM), peritoneal exudate (PEC) and lung. Instead more exosomes are retained in the blood (Fig.1G), which could indicate a requirement for Tspan8 to leave the blood stream. After repeated ifp application, recovery in lymphoid organs, lung and liver was reduced in rats receiving ASML-CD151^kd^ and/or -Tspan8^kd^ exosomes. Recovery of ASML-Tspan8^kd^ and –CD151/Tspan8^kd^ exosomes being particularly poor in the blood (Fig.1H), confirms the Tspan8 engagement in crossing the blood barrier. Counterstaining with leukocyte markers revealed, as described [52], that all leukocyte subpopulations, but most pronounced Mφ and DC take up exosomes. The uptake of ASML-CD151^kd^ and -Tspan8^kd^ exosomes is slightly and that of -CD151/Tspan8^kd^ exosomes is more severely impaired, which accounts for all leukocyte subpopulations. Notably, all leukocytes that uptake ASML exosomes are CD53^+^, which suggests a particular engagement of CD53 in exosome uptake by hematopoietic cells of the rat (Supp.Fig.3).

To obtain a hint, whether exosomal CD151 and Tspan8 affect premetastatic organ preparation, rats receiving poorly metastatic ASML-CD151/Tspan8^kd^ cells intrafoodpad (ifp) were pretreated with ASML^wt^, -CD151^kd^ or -Tspan8^kd^ exosomes. Exosome application (200μg/rat, ifp) was repeated every 3rd day. Rats were sacrificed 14 days after tumor cell application. The presence of ASML-CD151/Tspan8^kd^ cells was evaluated by flow cytometry in draining LNs, lung and BM. Except in rats receiving ASML^wt^ exosomes, tumor cells were hardly recovered particularly in lung and BM, indicating that both exosomal Tspan8 and CD151 contribute to niche preparation (Fig.1I).

Nonetheless, CD151- and Tspan8-competent exosomes most efficiently restored metastatic settlement of poorly metastatic ASML-CD151/Tspan8^kd^ cells. Thus, we asked for the contribution of exosomal tetraspanins.

### Exosomal CD151 and Tspan8 account for matrix remodelling

ASML cells express the tetraspanins CD151, Tspan8, CD9 and CD81. These tetraspanins are recovered in exosomes and the deficit of exosomal CD151 and/or Tspan8 has no significant impact on expression of the remaining tetraspanins in cells and exosomes (Fig.2A,2B). The major integrins in ASML are CD49c/(CD29), CD49f/CD104 that is recognized by the B5.5 antibody and, though less pronounced CD11b. Integrin expression is not affected in ASML-CD151^kd^, -Tspan8^kd^ and -CD151/Tspan8^kd^ cells, but CD11b and CD49c expression is reduced in ASML-CD151^kd^ and -CD151/Tspan8^kd^ exosomes and CD104 expression is reduced in ASML-Tspan8^kd^ and -CD151/Tspan8^kd^ exosomes (Fig.2C,2D). Reduced CD11b and CD49c expression in CD151^kd^ exosomes correlates with the preferential association of CD151 with CD11b and CD49c; strongly reduced recovery of CD104 in ASML-Tspan8^kd^ and -CD151/Tspan8^kd^ exosomes correlates with co-immunoprecipitation of CD104 with Tspan8 (Fig.2E), also described for ASML cells [53,54].

Despite reduced integrin expression, adhesion of exosomes to matrix proteins was not severely affected by a CD151 or Tspan8 deficit, but adhesion of ASML-CD151/Tspan8^kd^ exosomes was mitigated (Fig.2F). Blocking exosome adhesion by anti-CD49c or anti-CD104 confirmed a pronounced contribution of CD151 to CD49c-promoted adhesion and of Tspan8 to CD104-supported adhesion. Though differences did not reach statistical significance in all instances, the failure to block ASML-CD151/Tspan8^kd^ exosome adhesion by either anti-CD49c or anti-CD104 confirms the importance of the tetraspanin-associated integrins in exosome targeting (Fig.2G).

Exosome binding to matrix proteins is accompanied by matrix degradation, which is affected in ASML-CD151^kd^, -Tspan8^kd^ and -CD151/Tspan8^kd^ exosomes. ASML-CD151^kd^ exosomes did not degrade coll I and FN and poorly degraded coll II and coll IV. Coll II, FN and more pronounced LN332 degradation was reduced in the presence of ASML-Tspan8^kd^ compared to ASML^wt^ exosomes (Fig.3A). Reduced matrix degradation corresponds to reduced protease expression in exosomes. A CD151^kd^ mostly affects expression of MMP2 and MMP3, a Tspan8^kd^ affects MMP9, but promotes MMP14 and TACE expression. Furthermore, while exosomal MMP9 and MMP14 expression corresponds to cellular expression, MMP2, CD13 and TACE expression is reduced in ASML-Tspan8^kd^ exosomes (Fig.3B,3C). Notably, recovery in exosomes correlates with the tetraspanin association. Thus, after mild exosome lysis, CD13 (mostly), MMP9 and TACE co-immunoprecipitate with Tspan8, but not CD151. MMP2 and MMP14 co-immunoprecipitate with CD151, but not Tspan8 and exosomal MMP13 does not co-immunoprecipitate with CD151 or Tspan8, which was confirmed in the reverse setting in cell and exosome lysates for MMP2 and MMP9 (Fig.3D,3E). Furthermore, MMP2 was not recovered in light sucrose gradient fractions of ASML-CD151^kd^ exosomes, and MMP9, CD13 and TACE were not or poorly recovered in light sucrose gradient fractions of ASML-Tspan8^kd^ exosomes (Fig.3F). Thus, changes in the exosomal versus the cellular protease profile are due to the selective association of CD151 with MMP2 and MMP14 and of Tspan8 with CD13, MMP9 and TACE. Zymography confirmed the absence of active MMP2 in CD151^kd^ exosomes, strongly reduced MMP9 activity in ASML-Tspan8^kd^ exosomes and poor recovery of active MMP2 and MMP9 in CD151/Tspan8^kd^ exosomes (Fig.3G). The impact of CD151- and/or Tspan8-associated exosomal proteases also was apparent in degradation of the natural matrix of LnStr and LuFb. ASML-CD151^kd^ exosomes did not degrade coll I and LN111 and ASML-Tspan8^kd^ exosomes did not degrade LN332 (Fig.3H). Confirming the contribution of Tspan8-associated MMP9 and MMP13 to coll II, FN and LN332 modulation, degradation was abolished in the presence of an MMP9/MMP13 inhibitor, while degradation of coll I, coll IV and FN, which require CD151-associated MMP2, was not seen in the presence of an MMP2 inhibitor. LN332, coll IV and FN degradation was also impaired in the presence of the TACE inhibitor TAPI (Fig.3I).

**Figure 4:**
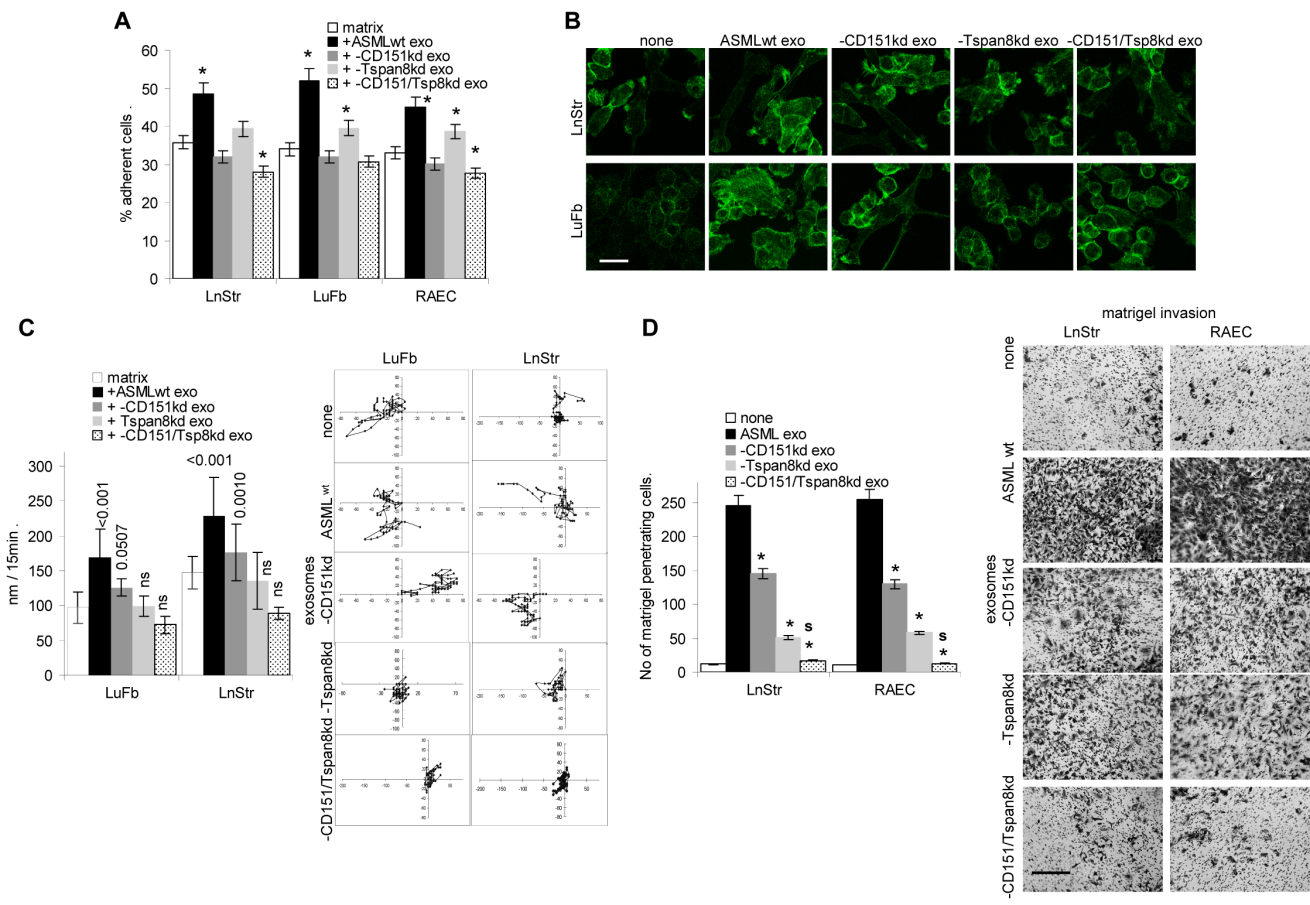
The impact of exosomal CD151 and Tspan8 on host cell adhesion, motility and invasiveness (A) LnStr, LuFb and RAEC were seeded on their corresponding untreated or exosome-treated matrix; mean±SD of adherent cells; significant differences compared to the native matrix: *; (B) confocal microscopy of actin cytoskeleton organization in LnStr and LuFb depending on exosome treated stroma (scale bar: 10μm); (C) videomicroscopy of LuFb and LnStr that were seeded on their untreated or ASML exosome-treated matrix. The relative migration of cells during 12h incubation (mean of 20 individual cells) and a representative example are shown; significant differences in migration due to ASML exosome treatment are indicated; (D) Matrigel penetration and invasion of LnStr and RAEC in dependence on CD151- and/or Tspan8-competent exosomes; the mean±SD (triplicates) of penetrating cells; significant differences to untreated matrigel: *; significant difference between ASML-CD151/Tspan8^kd^ versus -CD151^kd^ or -Tspan8^kd^ exosomes: s; and representative examples of invasion (scale bar: 250μm). Exosomal CD151 and Tspan8 slightly affect host cell adhesion to matrix proteins, but support stroma cell motility and invasiveness, which might be promoted by exosomal proteases.

Exosomal tetraspanin-integrin and tetraspanin-protease complexes also modulate the host matrix. Thus, lymph node stroma (LnStr), lung fibroblasts (LuFb) and rat endothelial cells (RAEC) adhere more strongly to their own matrix, when modulated by ASML^wt^ exosomes. Pronounced adhesion is not seen after pretreatment with ASML-CD151^kd^ or -CD151/Tspan8^kd^ exosomes (Fig.4A). Adhesion of stroma cells to the ASML^wt^ exosome-modulated matrix is accompanied by actin reorganization [51], which is also seen, though weaker after treatment with ASML-CD151^kd^ exosomes (Fig.4B). Furthermore, ASML^wt^ and -CD151^kd^ exosome-treated host matrices slightly promoted host cells motility, even though the effect was far weaker than on tumor cell motility (see Suppl.Fig.2B). Matrices treated with exosomes from Tspan8^kd^ or CD151/Tspan8^kd^ exosomes did not significantly affect host cell motility (Fig.4C). Finally, the deficit of protease activity in CD151^kd^ and/or Tspan8^kd^ exosomes had severe consequences on host cell invasiveness such that LnStr and RAEC poorly invaded matrigel, invasiveness being about 5-fold reduced in matrigel pretreated with ASML-CD151/Tspan8^kd^ exosomes compared to ASML^wt^ exosomes (Fig.4D).

Taken together, exosomal CD151 and Tspan8 account for integrin and protease recruitment [46-51], which results in matrix modulation [55]. Our findings fit with ASML^wt^ exosomes restoring metastatic capacity of ASML-CD151/Tspan8^kd^ cells as well as the inefficacy of ASML-CD151/Tspan8^kd^ exosomes to do so.

Besides modulating the extracellular matrix, tumor exosomes contribute to reprogramming host cells in premetastatic organs [11,56,57] and can affect the hematopoietic system [10,52,58-61]. Finally, tumor exosomes can affect neighboring tumor cells [62]. As exosomal tetraspanin complexes are essential for exosome binding and uptake [32], exosomes from ASML-CD151^kd^ and/or -Tspan8^kd^ cells now allowed us to ask for a genuine contribution of CD151 and Tspan8.

### Exosomal CD151 and Tspan8 and host cell modulation

Based on previous work demonstrating a major contribution of ASML exosomes to stroma cell protease and adhesion molecule as well as chemokine and chemokine receptor expression [63], we asked for a selective contribution of exosomal CD151 and Tspan8 in LnStr, LuFb and RAEC as well as LNC and BMC modulation. Exosomes were cocultured with LnStr, LuFb, RAEC and freshly harvested BMC and LNC for 48h.

**Figure 5:**
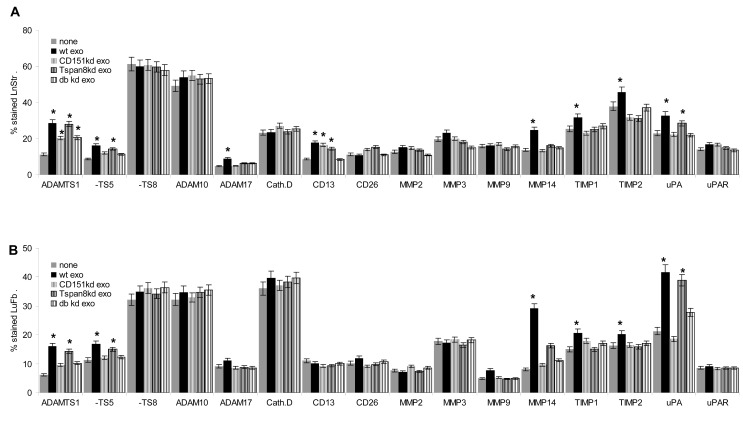

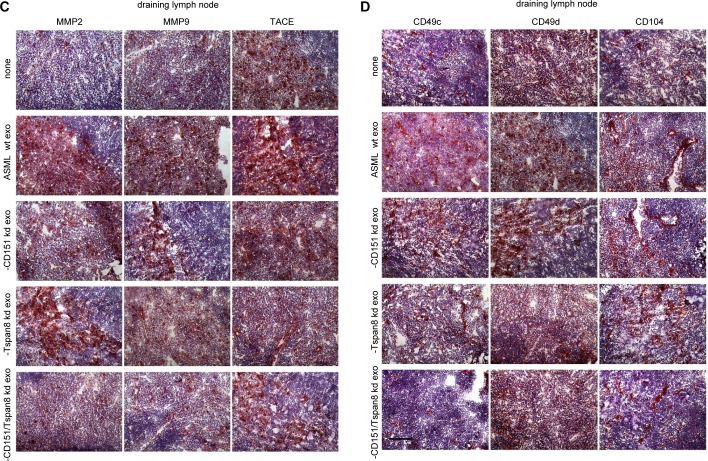
The impact of exosomal CD151 and Tspan8 on host cell adhesion molecule and protease expression (A,B) Flow cytometry analysis of protease expression in LnStr and LuFb after coculture with ASML^wt^, -CD151^kd^ and/or -Tspan8^kd^ exosomes; mean percent±SD (3 assays) of stained cells; significant differences to untreated cells: *; (C,D) immunohistology of protease and adhesion molecule expression in draining LN after repeated ifp application of ASML^wt^ or -CD151^kd^ and/or -Tspan8^kd^ exosomes (scale bar: 150μm). Short term *in vitro* coculture of stroma cells with ASML exosomes hardly affected protease and adhesion molecule (data not shown) expression. Instead, after repeated exosome application *in vivo*, ASML^wt^ exosomes particularly promoted MMP2, MMP9 and TACE as well as CD49c, CD49d and CD104 expression. CD104 and TACE upregulation were weakened in ASML-CD151/Tspan8^kd^ exosome treated rats and CD49c, CD49d, MMP2 and MMP9 expression were not supported.

We first controlled the impact of exosomal CD151 and Tspan8 on protease expression. Only ASML^wt^ exosome uptake by LuFb and more pronounced by LnStr promoted TACE, MMP14, TIMP1 and TIMP2 expression. ADAMTS1, ADAMTS5 and uPA upregulation appeared largely CD151-dependent. Expression of MMP2 and MMP9 was not affected by short term *in vitro* coculture (Fig.5A,5B). However, distinct to the short term *in vitro* coculture, repeated ASML exosome application promoted TACE, but also MMP2 and MMP9 upregulation that was strongest in ASML^wt^ exosome-treated rats. ASML-CD151/Tspan8^kd^ exosomes did not induce MMP9 and TACE upregulation; MMP2 expression was hardly increased in ASML-CD151^kd^ exosome- and MMP9 and TACE expression in -Tspan8^kd^ exosome-treated rats (Fig.5C). These findings fitted to the appearance of LN metastases with poor recovery of MMP2 in ASML-CD151^kd^ tumors, poor recovery of MMP9 in both ASML-CD151^kd^ and ASML-Tspan8^kd^ tumors and very few ADAM17^+^ cells in ASML-Tspan8^kd^ tumors. The reduced protease recovery was accompanied by a dense coll IV and LN332 matrix in ASML-CD151/Tspan8^kd^ tumors (Suppl.Fig.4). Immunohistology of LN sections from ASML^wt^ exosome-treated rats additionally showed upregulated expression of CD49c and CD49d that was not seen in ASML-CD151/Tspan8^kd^ exosome-treated rats. Upregulation of α6β4 was only seen in ASML^wt^ and ASML-CD151^kd^ exosome-treated rats (Fig.5D).

Thus, ASML exosomes exert a strong impact on host cell protease expression that partly depended on exosomal CD151 or Tspan8.

**Figure 6:**
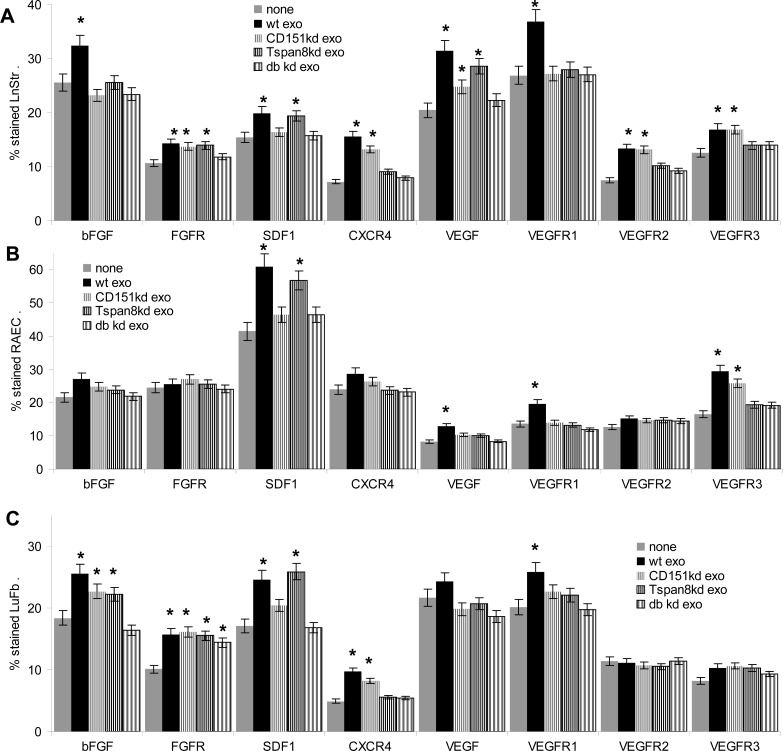

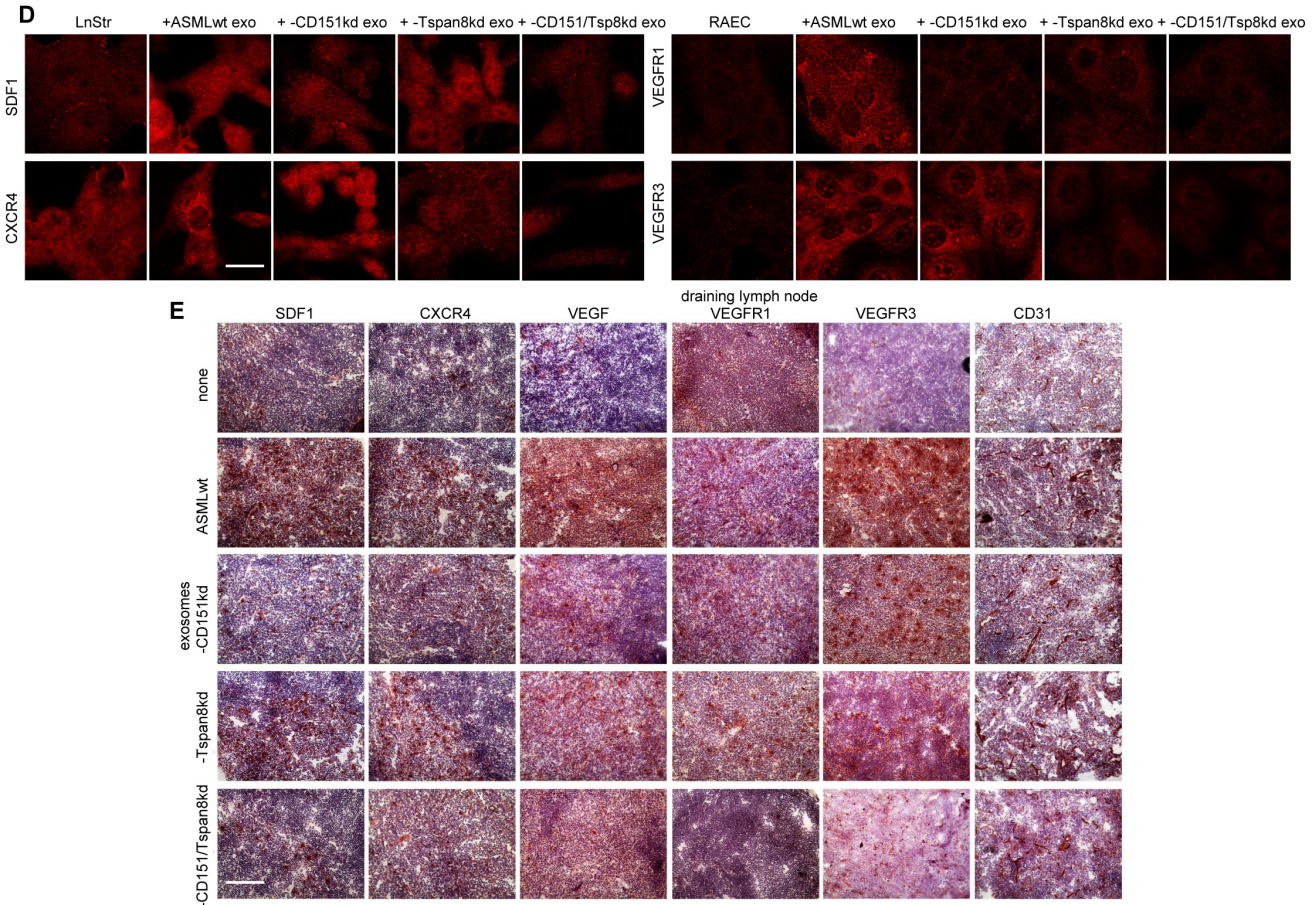
Stroma and endothelial cell responses to exosomal CD151 and Tspan8 (A-C) Flow cytometry analysis of LnStr, RAEC and LuFb after coculture with ASML^wt^, -CD151^kd^ and/or -Tspan8^kd^ exosomes; mean percent±SD (3 assays) of stained cells; significant differences to untreated cells: *; (D) representative examples of SDF1, CXCR4, VEGFR1 and VEGFR3 expression in LnStr and RAEC after coculture with ASML^wt^, -CD151^kd^ and/or -Tspan8^kd^ exosomes (scale bar: 10μm) and (E) in draining LN after repeated application of ASML^wt^, -CD151^kd^ and/or -Tspan8^kd^ exosomes (scale bar: 150μm). Exosomal CD151 and Tspan8 promote upregulated expression of several growth factors and their receptors, which varies depending on the target cell. Upregulated expression of some markers, e.g. bFGF and VEGFR1 essentially depends on the presence of both CD151 and Tspan8, whereas SDF1 and FGFR expression is independent of exosomal CD151 and Tspan8. However, exosomal Tspan8 is essential for VEGFR3 upregulation.

Premetastatic niches are frequently characterized by changes in chemokines and their receptors [64], where we controlled for a selective impact of exosomal CD151 and Tspan8 in LnStr, RAEC and LuFb after 48h coculture with ASML^wt^, -CD151^kd^ and/or -Tspan8^kd^ exosomes. LnStr, LuFb and RAEC did not uniformly respond to ASML exosome treatment. Only SDF1 and VEGFR1 were upregulated in all three cell populations after treatment with ASML^wt^ exosomes. However, SDF1 depended more on CD151 than Tspan8, whereas VEGFR1 stimulation required CD151 and Tspan8. CXCR4 and VEGFR3 expression, stimulated in LnStr and LuFb, respectively in LnStr and RAEC, required Tspan8-competent exosomes. FGFR upregulation in LnStr and LuFb was independent of exosomal CD151 and Tspan8 (Fig.6A-6D). These findings could be confirmed by staining LN of ASML exosome-treated rats (Fig.6E).

Taken together, a strong induction of cytokine/chemokine and -receptor expression, most notably of VEGFR3 in RAEC as well as of CXCR4 both depend on Tspan8.

**Figure 7:**
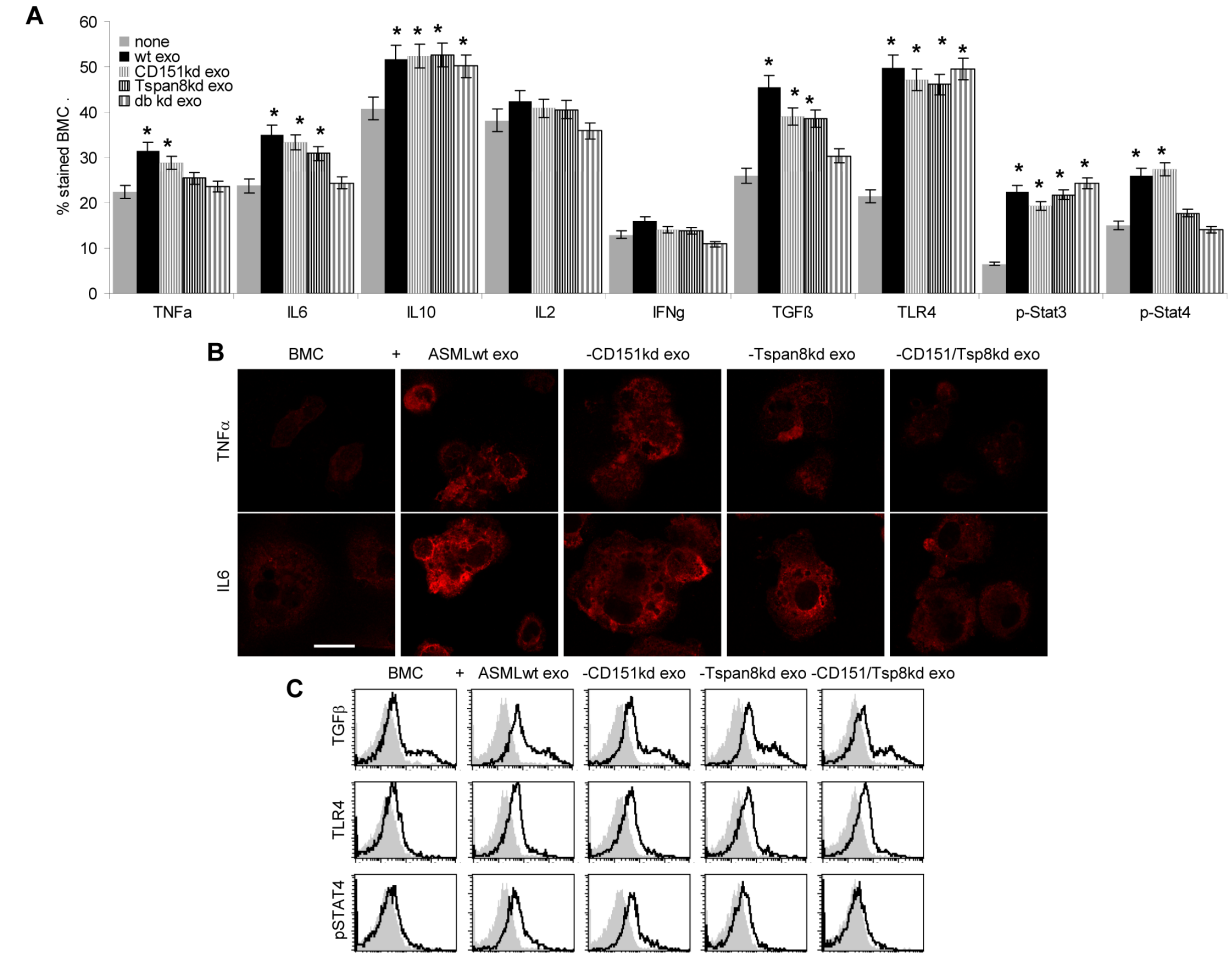

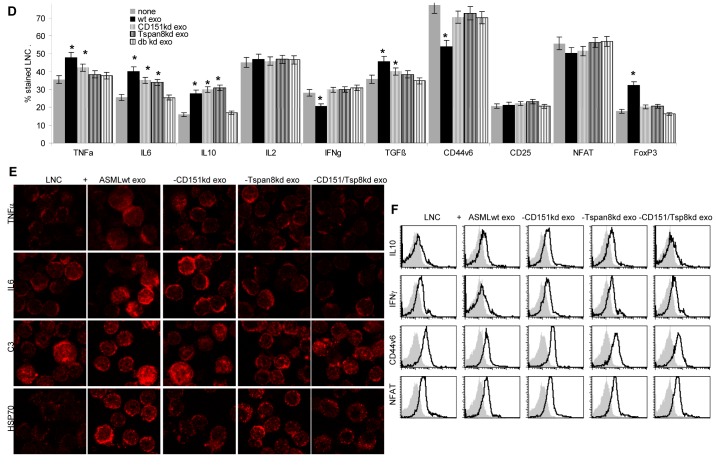
Leukocyte responses to exosomal CD151 and Tspan8 (A-F) BMC and LNC were cocultured with ASML^wt^, -CD151^kd^ and/or -Tspan8^kd^ exosomes; (A,C,D,F) cytokine and activation marker expression as well as activation of the JAK/STAT signaling pathway and of NFAT and FoxP3 was evaluated by flow cytometry; mean percent±SD of stained cells; significant differences to BMC and LNC cultured in the absence of exosomes: * and representative examples; (B,E) representative examples of confocal microscopy (scale bar: 10μm). In BMC and LNC, ASML exosomes strengthen inflammatory cytokine expression; These effects are supported by, but not exclusively depending on exosomal CD151 and Tspan8.

Tumor exosomes were reported to promote inflammation and immunosuppression [59-61,65]. Indeed, inflammatory TNFα and IL6 expression became upregulated in BMC and LNC. The latter also expressing C3 and HSP70 at an increased level. We also noted upregulated expression of the immunosuppressive cytokine IL10, but expression of the effector cytokines IL2 and IFNγ as well as of the activation markers CD44v6 and CD69 were unaltered or slightly reduced only in ASM^wt^ exosome treated LNC. Upregulation of TGFβ was more pronounced in BMC than LNC cocultures, where it should be noted that ASML exosomes more strongly affected BMC than LNC. Confirming the exosome-induced shift towards inflammation / immunosuppression, TLR4, p-Stat3 and p-Stat4 expression were upregulated in BMC and LNC (data not shown) and FoxP3 expression was increased in LNC, but NFAT expression was not altered. Furthermore, while ASML-CD151/Tspan8^kd^ exosomes hardly exerted any effect, in most instances ASML^wt^ exosomes had the strongest impact (Fig.7A-7F).

In brief, there was a shift towards inflammatory and immunosuppressive cytokines without evidence for significantly impaired effector cytokines. The stronger impact on BMC than LNC and the missing effect of ASML-CD151/Tspan8^kd^ exosomes are in line with the more ready uptake of ASML exosomes by BMC and the poor uptake of ASML-CD151/Tspan8^kd^ exosomes (Suppl.Fig.3). Furthermore, though changes were most pronounced after coculture with ASML^wt^ exosomes, they were not abolished after coculture with -CD151^kd^ or -Tspan8^kd^ exosomes, indicating that the major contribution of exosomal tetraspanins may rely on binding rather than distinct message transfer.

### Exosomal CD151 and Tspan8 and the feedback towards non-metastasizing tumor cells

As exosomes itinerate [66], tumor exosomes could well affect neighboring tumor cells, such that tumor stem cell exosomes modulate non-CIC.

The CD151^kd^ and/or Tspan8^kd^ had no major impact on receptor tyrosine kinase (RTK) expression with the exception of VEGFR1, which is weakly expressed in ASML^wt^ cells, expression being further reduced in ASML-CD151^kd^ and ASML-Tspan8^kd^ cells (Suppl.Fig.5A). When ASML-CD151/Tspan8^kd^ cells were cocultured with ASML^wt^ exosomes, VEGFR1 and, predominantly, VEGFR3 expression became upregulated (Suppl.Fig.5C-5E). As expression in exosomes was low (Suppl.Fig.5B), a direct protein transfer can be excluded [63].

**Figure 8:**
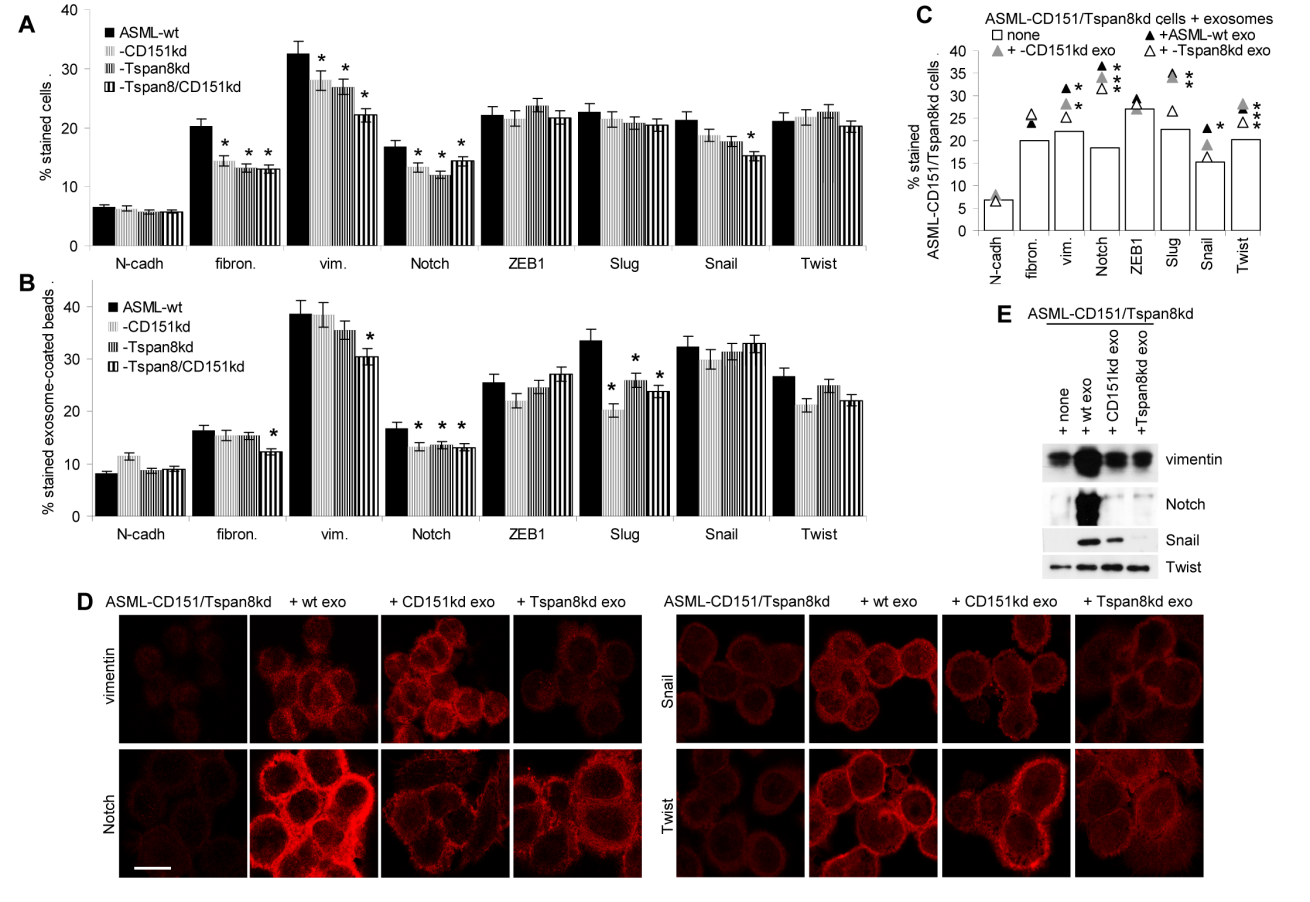
Metastasis-supporting exosomes and EMT: ASML-CD151/Tspan8^kd^ cells were cocultured for 48h with ASML^wt^, -CD151^kd^ or -Tspan8^kd^ exosomes (A,B) ASML^wt^, -CD151^kd^ and/or -Tspan8^kd^ cells and exosomes stained for the indicated EMT markers; mean percent±SD of stained cells / exosome-coated latex beads; significant differences to ASML^wt^ cells / exosomes: *. (C-E) EMT gene expression evaluated by flow cytometry (mean percent stained cells, 3 assays); significant differences to ASML-CD151/Tspan8^kd^ cells cultured in the absence of exosomes: *, confocal microscopy (scale bar: 10μm) and WB. Expression of the EMT related vimentin protein as well as of the transcription factors Slug, Snail, Twist and, particularly, Notch, becomes upregulated, upregulation of vimentin and Snail predominantly depending on Tspan8 and of Notch on CD151 and Tspan8.

In concern of EMT-related factors including EMT-associated transcription factors, only FN, vimentin, Notch and, borderline, Snail were downregulated in ASML-CD151^kd^ and/or ASML-Tspan8^kd^ cells. In exosomes, only expression of Slug and, slightly of Notch was reduced by the CD151^kd^ and/or the Tspan8^kd^ (Fig.8A,8B). Instead, after coculture of ASML-CD151/Tspan8^kd^ cells with exosomes, expression of vimentin, Slug, Twist and, most strikingly, Notch became upregulated. While vimentin, Snail and Slug appeared to depend mostly on Tspan8, the strong upregulation of Notch required CD151- and Tspan8-competent exosomes (Fig.8C-8E).

Coculture of poorly metastatic ASML-CD151/Tspan8^kd^ cells with ASML^wt^ exosomes promoting upregulation of several EMT-related proteins and transcription factors supports the hypothesis that exosomes from metastasizing tumor cells can drive neighboring non-metastatic tumor cells into EMT.

In brief, the metastasis-supporting activity of the tetraspanins CD151 and Tspan8 most likely is promoted by exosomes, which strongly modulate the host matrix, affect hematopoietic and stroma cell activation including (lymph)angiogenesis. In addition, the transfer of exosomes into neighboring non-metastatic tumor cells supports expression of EMT-related genes.

## DISCUSSION

Exosomes are important intercellular communicators [14,15] and it was suggested that tumor exosomes, particularly cancer initiating cell exosomes account for premetastatic niche preparation [9-11]. Although the mechanisms are not yet elucidated, it is evident that exosomal targeting structures as well as transferred proteins and most prominently transferred miRNA account for the reprogramming of host cells towards supporting settlement and growth of disseminated tumor cells [9]. As tetraspanins are constitutive exosome components and known to be engaged in exosome binding and uptake [32], we speculated that the metastasis-promoting tetraspanins CD151 and Tspan8 [25,26,37-40] could play a central role in the process of tumor exosome-mediated host modulation. We demonstrate that ASML-CD151/Tspan8^kd^ cells largely lost the capacity to metastasize, but regain metastatic capacity by the support of ASML^wt^ exosomes. Deciphering the role of exosomal CD151 and Tspan8 revealed that both tetraspanins contribute to matrix degradation that supports tumor and host cell motility, affect stroma and hematopoietic cells and are engaged in a feedback towards non-metastatic tumor cells, where they promote EMT.

### Loss of metastasis formation by a double knockdown of CD151 and Tspan8

In advance of evaluating the impact of exosomal tetraspanins on the crosstalk with the host, we reassured that ASML-CD151/Tspan8^kd^ cells lost metastatic potential. The *in vivo* and *in vitro* growth characteristics of ASML-CD151/Tspan8^kd^ cells resembled the sum of that reported for ASML-CD151^kd^ and ASML-Tspan8^kd^ cells [51]. Similar to ASML-Tspan8^kd^ cells, anchorage independent growth of ASML-CD151/Tspan8^kd^ cells was strongly impaired and apoptosis resistance was more severely affected than in ASML-CD151^kd^ or ASML-Tspan8^kd^ cells (data not shown). Migration was similar to that of ASML^wt^ cells, i.e. the striking gain in motility seen in ASML-CD151^kd^ cells was annulated. ASML-CD151/Tspan8^kd^ cells were poorly invasive and scarce transendothelial cell migration resembled that of ASML-Tspan8^kd^ cells without a measurable contribution of CD151. These findings, which confirmed our results on ASML-CD151^kd^ and ASML-Tspan8^kd^ cells, provided a solid basis to answer the question on the role of exosomal CD151 and Tspan8 in metastasis.

### Exosomal CD151 and Tspan8 and the host matrix

Exosomes maintain the tetraspanin webs of their cells of origin [24], the presented data confirming the general validity of TEM complex recovery in exosomes. Thus, the tetraspanin – integrin associations are maintained in exosomes. Reduced expression of either CD151 or Tspan8 apparently is not sufficient to significantly alter exosome-binding to matrix proteins. This includes the adhesion promoting, but migration inhibitory effect of CD151 that was visible, but less pronounced in exosomes than cells [51]. Instead, the exosomal tetraspanin-protease complexes displayed full activity with a strong impact on invasion, where matrix degradation became strongly impaired or abolished by a deficit of the tetraspanin partner for proteases. Thus, a deficit in CD151 was accompanied by a failure to degrade coll I or FN and degradation of coll IV was reduced, which correlated with poor recovery of CD151-linked MMP2 in ASML-CD151^kd^ cells. Due to the preferential association of MMP9 with Tspan8, ASML-Tspan8^kd^ exosomes poorly degraded coll I, coll IV and LN332 and degradation was abolished in the presence of an MMP9 inhibitor. The TACE inhibitor TAPI impaired LN332 and partly coll II, coll IV and FN degradation, which could be due to the preferential, though not exclusive association with Tspan8. Importantly, exosomal tetraspanin-associated proteases modulate stroma matrices *in vivo* such that invasion is facilitated. This became most obvious for Tspan8 upon iv and ifp application of dye-labeled exosomes, where Tspan8^kd^ and CD151/Tspan8^kd^ exosomes were retained in the blood after iv and poorly recovered in the blood after ifp application, which fitted to the deficit of ASML-Tspan8^kd^ and -CD151/Tspan8^kd^ cells to pass an endothelial cell monolayer and previous reports on Tspan8 preferentially targeting endothelial cells [32,67].

In brief, the crosstalk between tumor exosomes and the matrix is strongly influenced by exosomal tetraspanins. Due to their associations with integrins and proteases, the tetraspanin web facilitates binding, motility and matrix degradation.

### Stroma and hematopoietic cell modulation by exosomal CD151 and Tspan8

Coculture of LnStr, LuFb, RAEC and freshly harvested BMC or LNC with ASML^wt^, -CD151^kd^, -Tspan8^kd^ and –CD151/Tspan8^kd^ exosomes allowed appointing changes in target cells to be CD151- and/or Tspan8-dependent or -independent. However, it should be remembered that there is strong evidence that exosomes modulate target cells mostly via transferred miRNA [63]. As we have not yet explored the impact of CD151 and Tspan8 on miRNA recruitment into exosomes, we presently cannot differentiate between exosomal CD151- and Tspan8-mediated effects due to tetraspanin binding versus a potential impact due to transferred messages. Independent of this open question, exosomal CD151 and Tspan8 significantly contribute to target cell modulation, where we concentrated on adhesion molecules, proteases, chemokines and their receptors and, particularly in BMC and LNC on changes associated with immunosuppression and inflammation.

Exosome uptake significantly affected adhesion molecule and protease expression in lymph nodes / lymph node stroma cells and lung / lung fibroblasts. With few exceptions, ASML^wt^ exosomes exerted the strongest effect, which we interpret in the sense that a major contribution of CD151 and Tspan8 might rely on facilitating binding and uptake. Notably, effects on protease and adhesion molecule expression were mostly stronger after *in vivo* application than after *in vitro* coculture. We consider *in vivo* cooperation between exosome target cells and the exosome modulated matrix as a likely explanation.

Expression of several chemokines and angiogenesis-related factors and -receptors were significantly affected by ASML^wt^ exosomes. Differences in the response to ASML^wt^ versus -CD151^kd^, -Tspan8^kd^ and -CD151/Tspan8^kd^ exosomes provided additional evidence for a selective engagement of exosomal CD151 in SDF1 upregulation and of exosomal Tspan8 in VEGFR2, VEGFR3 and CD31 upregulation. In cooperation with exosomal CD49d Tspan8 initiates overshooting angiogenesis [67-69]. Instead, ASML^wt^ cells and exosomes, which do not express CD49d, selectively support lymphatic metastasis [70], fitting the upregulation of VEGFR3 [71]. Though many carcinoma metastasize via blood and lymphatic vessels, lymph nodes frequently are the first metastatic station [72]. Thus, the selective and efficient contribution of exosomal Tspan8 towards VEGFR3 induction deserves further studies to unravel the underlying mechanism, which may provide a hint towards therapeutic interference.

Tumor exosomes can be immunosuppressive [59], which frequently is accompanied by a shift towards immunosuppressive subpopulations like myeloid-derived suppressor cells and T_reg_ [11,58,59,73,74]. We noted, an increase in the inflammatory cytokines TNFα and IL6 and the immunosuppressive cytokine IL10 [75-77], however without evidence for significantly impaired effector cytokine expression. This is surprising as FoxP3 became strongly upregulated in LNC cocultured with ASML^wt^ exosomesand [78]. In LNC and, more pronounced in BMC TFGβ, a central regulator of immune response [78,79], was upregulated. In line with this finding, ASML exosomes promoted JAK/Stat activation [76-78]. The impact of exosomes on inflammatory cytokines and TGFβ being more pronounced in BMC, which very efficiently take up tumor exosomes, and being strongest after coculture with ASML^wt^, but hardly seen with poorly binding ASML-CD151/Tspan8^kd^ exosomes points towards exosomal CD151 and Tspan8 particularly contributing to exosome binding/uptake.

Taken together, tumor exosomes support induction of an inflammatory response and stroma cell activation. The engagement of exosomal CD151 and Tspan8 in inflammation mostly relies on facilitating binding and uptake. Instead, exosomal CD151 and/or Tspan8 have bearing on SDF1, CXCR4, VEGFR1 and, most relevant for lymphatic metastasis, VEGFR3 expression, such that a blockade of tetraspanins could well provide a therapeutic option. This consideration gains weight by the feedback of exosomes from metastasizing on non-metastatic tumor cells.

### The contribution of exosomal CD151 and Tspan8 to epithelial mesenchymal transition

Metastasizing tumor cells are supposed to present a subpopulation of CIC [80] and it has been argued that CIC transfer the potential to metastasize to non-metastatic tumor cells possibly via exosomes [81-83]. Thus, we finally controlled the impact of ASML^wt^ exosomes on ASML-CD151/Tspan8^kd^ cells. RTK expression remained unaltered except for VEGFR1 and VEGFR3. Expression being very low in ASML cells and exosomes excludes changes by coculture with ASML^wt^ exosomes to be due to direct protein transfer. The functional relevance of VEGFR expression in tumor cells as well as the pathway, whereby exosomes account for VEGFR expression, also seen in non-transformed host cells, remains to be explored. Lymphangiogenesis regulation is a complex process, which involves Ras, the MAPK cascade, PI3K/Akt signaling, TGFβ and Notch [71], where our data point towards a special engagment of Notch.

In view of the partial overlap of (lymph)angiogenesis and EMT [84] and being aware that EMT is one of the first steps towards metastasis [85,86], we evaluated EMT gene expression in ASML-CD151/Tspan8^kd^ cells after coculture with ASML^wt^ and for comparison -CD151^kd^ and -Tspan8^kd^ exosomes. ASML^wt^ exosomes most strongly upregulated Notch. The intracellular domain of Notch binds to RBP-Jκ (recombination binding protein-Jκ), converting the repressor complex into an activator of Notch target gene transcription [87]. Thus, it is tempting to speculate that exosomal proteins and/or miRNA affect a central, upstream component in Notch signaling, as vimentin, Snail, Slug and Twist expression also became upregulated, though to a minor degree. Similar to the regulation of VEGFR3, the impact of ASML-Tspan8^kd^ exosomes was weaker than that of ASML^wt^ exosomes, which we interpret as indicating a contribution of exosomal tetraspanins, but also excluding the tetraspanins as exclusive mediators.

Thus, ASML^wt^ exosomes promote EMT in non-metastatic cells. The striking impact of exosomal CD151 and Tspan8 demands for elucidating the underlying mechanism, where we presently cannot exclude a major contribution by tetraspanin-facilitated exosome binding and uptake. Despite this open question, induction of EMT by exosomes of a metastasizing tumor line in a non-metastasizing tumor line strongly supports our hypothesis on the central importance of exosomal tetraspanins in promoting metastasis.

## CONCLUSION

We here provide strong evidence for a central role of exosomal tetraspanins in metastasis. Exosomes facilitate motility and invasiveness by modulating the extracellular matrix, a process wherein exosomal CD151 and Tspan8 are directly engaged due to their associations with integrins and proteases. Exosomes also drive hematopoietic cells towards an inflammatory phenotype and initiate protease and chemokine / chemokine receptor expression in stroma cells. Most importantly, exosomes stimulate EMT in non-metastatic cells. CD151 and Tspan8 are essential for exosome-initiated target cell and non-CIC activation and reprogramming. It remains to be explores, whether the tetraspanin contribution is a sequel of the engagement in exosome binding and uptake or whether tetraspanins additionally contribute to the recruitment of signaling molecules and/or miRNA. Irrespective of this open question, a blockade of exosomal Tspan8 and CD151 appears most promising for interfering with metastasis.

## MATERIAL AND METHODS

### Cell lines

ASML [70], ASML-Tspan8^kd^, ASML-CD151^kd^ [51], ASML-CD151/Tspan8^kd^, LN332 secreting 804G [88], LuFb, LnStr (B12) [89], RAEC lines were maintained in RPMI1640/10%FCS, supplemented for ASML-Tspan8^kd^ and ASML-CD151^kd^ cells with 750μg/ml G418 and for ASML-CD151/Tspan8^kd^ additionally with 5μg/ml puromycin. ASML-Tspan8^kd^ were transfected with pSuperGFP-neoplasmid containing CD151 shRNA to generate stable double knockdown lines (Primers: Tab.S1) (Qiagen, Hildesheim, Germany) following the supplier's suggestion.

### Antibodies

see Tab.S2.

### Cell and tissue preparation

The local tumor, draining lymph nodes, lung and bone marrow were collected. Single cell suspensions were obtained by pressing through fine gauze. Alternatively, organs were shock frozen or lysates were prepared by tissue disruption (UltraTurrax, 3-times, 30sec on ice).

### Exosome preparation

Cells were cultured (48h) in serum-free medium. Cleared supernatants (2×10min, 500g, 1×20min, 2000g, 1×30min, 10000g) were centrifuged (90min, 100000g) and washed (PBS, 90min, 100000g). The resuspended pellet was purified by sucrose gradient centrifugation. Where indicated, exosomes were SP-Dio_18_(3)-labeled before sucrose gradient centrifugation and 2 washings [24].

### Sucrose density gradient centrifugation

Cell lysates or exosomes in 2.5M sucrose were overlaid by a continuous sucrose gradient (0.25M-2M) and centrifuged (15h, 150000g), collecting twelve 1ml fractions.

Immunoprecipitation, Western blot: Lysates (HEPES buffer, 1%Lubrol or 1%Brij96, 1mM PMSF, protease inhibitor mix, 30min, 4°C) were immunoprecipitated, incubated with ProteinG-Sepharose (1h) and washed. Lysates/immunoprecipitates were resolved on 7-12% SDS-PAGE. After transfer, blocking, immunoblotting with primary and HRP-labeled Streptavidin or secondary antibodies, blots were developed with the ECL detection system.

Flow cytometry followed routine procedures. For intracellular staining, cells were fixed with 1% formalin and permeabilized (0.5% Tween in PBS) in advance. Apoptosis was determined by AnnV/PI staining. Exosomes were incubated with 4μm aldehyde-sulfate latex-beads (Invitrogen, Karlsruhe, Germany), blocking free aldehyde groups (PBS/100mM glycine, 20min, 20^o^C) before staining. Staining was evaluated using a FACS-Calibur and the Cell Quest program (BD, Heidelberg, Germany).

### Histology and immunofluorescence

Snap frozen sections (8μm) were fixed with chloroform/aceton (1:1), 4min, dried and incubated with antibodies, washed, exposed to biotinylated secondary antibodies and alkaline phosphatase conjugated avidin-biotin solution, counter-staining sections with H&E. Endogenous tissue alkaline phosphatase activity was ablated with levamisole solution and non-specific binding was blocked using an avidin-biotin blocking kit (Vector Laboratories, Burlingame, CA, USA). Immunofluorescence staining followed routine procedures. For intracellular staining cells were permeabilized with 0.1% Triton after fixation with 4% Formaldehyde. Slides were mounted in Elvanol to generate digitized images (Carl Zeiss LSM710 confocal microscope; software Carl Zeiss Axioview Rel. 4.6).

### Adhesion

Cells were seeded on matrix protein-coated 96-well F-bottom plates. After washing, adherent cells were stained with crystal-violet and lysed, evaluating OD595 photometrically. Adhesion is presented as the percentage of input cells.

### Migration

Cells, in the upper part of a Boyden chamber (RPMI/0.1%BSA), were separated from the lower part (RPMI/20%FCS) by 8μm pore size polycarbonate-membranes. After 16h the lower membrane side was stained (crystal-violet), measuring OD595 after lysis. For *in vitro* wound healing, a subconfluent monolayer was scratched with a pipette tip, following wound closure by light-microscopy. For video-microscopy, Hoechst 33342 stained cells (5×10^4^) were seeded on matrix-coated 24-well plates. Plates were placed under an Olympus IX81 inverse microscope with a Hg/Xe lamp, an incubation chamber (37^o^C, 5%CO_2_), a CCD camera (Hamamatsu) and a ScanR acquisition soft ware (Olympus, Hamburg, Germany). Two pictures (20-fold magnification) / chamber (2ms exposure) were taken every 20min for 12h. Migration was quantified according to Manual_tracking plugin running in the open-source software Image J.

### Invasion

Polycarbonate membranes (8μm pore size) transwell permeable supports were coated with 100μl 1:5 diluted Matrigel and kept at 37^o^C in a humidified atmosphere overnight [55]. After washing, 5×10^4^ cells in 200μl RPMI/1% BSA were placed on the gels. The lower chamber contained RPMI with 20% FCS. After 48h at 37^o^C, 5%CO_2_, the medium in the insert was removed and cells not invading the gels were washed off. Matrix invasion and recovery on the lower membrane side was evaluated microscopically and photometrically after crystal-violet staining and lysis.

### Proliferation

Cells (2×10^4^) were seeded in F-bottom 96-well plates adding ^3^H-thymidine (10μCi/ml). ^3^H-thymidine incorporation was evaluated after 24h (β-counter). Alternatively, cells were CFSE (carboxy fluorescein succinimidyl ester)-labeled, determining CFSE dilution after 24h, 48h and 72h by flow-cytometry.

Cell cycle progression was evaluated using PI staining and standard procedures.

### Apoptosis

Cells (1×10^5^) were grown for 48h in RPMI/10%FCS containing cisplatin. Survival was monitored by annexinV-APC/PI staining and ^3^H-thymidine uptake.

### Soft agar assay

Tumor cells in 0.3% agar were seeded in 6-well plates on a preformed 1% agar layer counting colonies after 3wk.

### Zymography

Conditioned medium (CM) of starved cells (1×10^6^) was centrifuged (15min, 15000g) and/or depleted of exosomes (CM^−exo^). Aliquots were incubated with Laemmli buffer (15min, 37^o^C) and separated in a 10% acrylamide gel containing 1mg/ml gelatin. After 3x washing (2.5% Triton), gels were incubated in developing buffer (50mM Tris, 10mM CaCl_2_, 150mM NaCl, 2μM ZnCl_2_, pH 7.5) (37^o^C, 48h) and stained with Coomassie-blue.

### *In vivo* assays

BDX rats received 1×10^6^ tumor cells, ifp. Where indicated, rats received concomitantly and repeated in 3 day intervals 200μg exosomes ifp. The local growth, growth in the popliteal and the inguinal lymph node were evaluated weekly with sliding calipers. Animals were sacrificed at the indicated time points or when draining nodes reached 2cm diameter, upon >10% weight loss, short breathing and a stiff thorax due to lung metastases or latest after 120d. For the evaluation of exosome distribution, rats received 200μg dye-labeled exosomes, iv or ifp. Animals were sacrificed at the indicated time points. They were bled by heart puncture and all lymphatic / hematopoietic organs were excised and analyzed by flow cytometry for the presence of labeled cells. Animal experiments were Government-approved (Baden-Wuerttemberg, Germany).

### Statistical analysis

Assays were repeated at least 3 times. P-values <0.05 (two-tailed Student's t-test, Anova) were considered significant.

### Authorship

SY, WM and UE performed and analyzed experiments, MZ planned and analyzed experiments and wrote the manuscript.

## SUPPLEMENTARYMATERIAL, TABLES AND FIGURES



## Abbreviations

CIC: cancer initiating cells
CM: conditioned medium
coll: collagen
EMT: epithelial-mesenchymal transition
FN: fibronectin
HA: hyaluronic acid
ifp: intrafootpad
iv: intravenously
kd: knockdown
LN: lymph node
LN111/LN332: laminin111/laminin332
LnStr: LN stroma line
LuFb: lung fibroblasts
RAEC: rat endothelial cell line
RTK: receptor tyrosine kinase
TEM: tetraspanin-enriched microdomain
wt: wild type
