# Supplementary material for: The tetraspanins CD151 and Tspan8 are essential exosome components for the crosstalk between cancer initiating cells and their surrounding
Source: Oncotarget. 2014 Dec 10;6(4):2366–84. doi: 10.18632/oncotarget.2958 (PMC4385857; doi:10.18632/oncotarget.2958)
Supplement: Supplementary file 1 [file oncotarget-06-2366-s001.pdf]

The tetraspanins CD151 and Tspan8 are essential exosome components for the crosstalk between cancer initiating cells and their surrounding

Supplementary Material

Table S1 **Primers, antibodies and chemicals**

Table S1A **Tspan8 and CD151 knockdown primers**

Tspan8 kd forward	5'-gatccccTGATTCTGCTTCTGCAAGTttcaagagaACTTGCAGAAGCAGAATCAttttta-3'
Tspan8 kd reverse	5'-agcttaaaaaTGATTCTGCTTCTGCAAGTtctcttgaaACTTGCAGAAGCAGAATCAggg-3'
CD151 kd forward	5'-gatccccCCTCAAGAGTGACTACATTttcaagagaAATGTAGTCACTCTTGAGGttttta-3'
CD151 kd reverse	5'-agcttaaaaaCCTCAAGAGTGACTACATTtctcttgaaAATGTAGTCACTCTTGAGGggg-3'

Tspan8 and CD151 knockdown sequences were cloned into the pSUPER-RNAi vector within the Bgl II and Hind III cleavage sites.

Table S1B **Antibodies**

**Antibody**	**Origin**	**Supplier**
actin	mouse	Becton Dickinson[b]
ADAM10	goat	Santa Cruz
ADAM17	rabbit	Stressgen
ADAMTS1	mouse	Santa Cruz
ADAMTS5	rabbit	Santa Cruz
ADAMTS8	goat	Santa Cruz
ankyrin	rabbit	Santa Cruz
Bcl2	mouse	Becton Dickinson
BclXl	mouse	Becton Dickinson
bFGF	mouse	Becton Dickinson
C4.4A	mouse	clone C4.4 (1)[a]
Casp3	rabbit	Becton Dickinson
Casp3, activated	rabbit	Becton Dickinson
Casp8	rabbit	Dianova
Casp9, cleaved	rabbit	Cell Signaling
CathepsinD	rabbit	Santa Cruz
CD9	mouse	clone B2C11 (DSHB)[b],BD
CD11b (αM)	mouse	clone Ox42 (EAACC)[b]
CD13	rabbit	Santa Cruz
CD29 (β1)	rabbit	Becton Dickinson
CD26	mouse	Becton Dickinson
CD31	mouse	Becton Dickinson
CD44	mouse	clone Ox50 (EAACC)
CD44v6	mouse	clone A2.6 (1)[a]
CD49c (α3)	mouse	clone Ralph3.1 (EAACC), BD
CD49d (α4)	mouse	Becton Dickinson
CD49e (α5)	hamster	Becton Dickinson
CD49f (α6)	rabbit	Santa Cruz
CD49f/CD104 (α6β4)	mouse	clone B5.5 (1)[a]
CD54	mouse	Becton Dickinson
CD81	hamster	Santa Cruz
CD104 (β4)	rabbit	Becton Dickinson
CD106	mouse	Becton Dickinson
CD151	rabbit	(2)[a]
coll I	rabbit	Rockland
coll II	rabbit	Dianova
coll IV	rabbit	Rockland
CXCR4 (CD184)	rabbit	Becton Dickinson

Table S1B continued

Antibody	Origin	Supplier
EGFR	rabbit	Biotrend
EpCAM	mouse	clone D5.7 (1)[a]
ezrin	rabbit	Sigma
FAK	rabbit	Becton Dickinson
FGFR	mouse	Biotrend
fibronectin	mouse	Becton Dickinson
Gr1	mouse	Becton Dickinson
IGFR	rabbit	Santa Cruz
IL6	rat	Becton Dickinson
JAK1	mouse	Becton Dickinson
LNβ1 (β1-chain)	rabbit	Becton Dickinson
LNγ2 (γ2-chain)	rabbit	Rockland
MET	rabbit	Becton Dickinson
MMP2	rabbit	Dianova
MMP3	rabbit	Abcam
MMP9	rabbit	Dianova
MMP13	rabbit	Dianova
MMP14	rabbit	Santa Cruz
mTOR	rabbit	Santa Cruz
N-cadherin	rabbit	Santa Cruz
Notch1	mouse	Biolegend
osteopontin	rabbit	Biolegend
p-Akt	rabbit	Biotrend
p-BAD	rabbit	Santa Cruz
p-ERK1/2	mouse	Becton Dickinson
p-JNK	mouse	Becton Dickinson
p-jun	mouse	Santa Cruz
p-p38	mouse	Becton Dickinson
p-paxillin	rabbit	Cell Signaling
p-PKC	mouse	Cell Signaling
p-PI3K	mouse	Santa Cruz
p-PLCγ1	mouse	Cell Signaling
PP1a	rabbit	Cell Signaling
p-PP1a	rabbit	Cell Signaling
PP2a	mouse	Santa Cruz
p-Rac	rabbit	Cell Signaling
p-Ras	mouse	Becton Dickinson
p-src	rabbit	Cell Signaling
paxillin	rabbit	Becton Dickinson
PDFGR	rabbit	Santa Cruz
p-STAT3	mouse	Becton Dickinson
p-STAT4	mouse	Becton Dickinson
Pten	mouse	Becton Dickinson
Rac	rabbit	Cell Signaling
Ras	mouse	Becton Dickinson
RhoA	mouse	Becton Dickinson
SDF1	rabbit	abcam
Slug	rabbit	Santa Cruz
Snail	rabbit	Santa Cruz
src	rabbit	Santa Cruz
STAT3	mouse	Becton Dickinson
STAT4	mouse	Becton Dickinson
STAT6	mouse	Becton Dickinson

Table S1B continued

**Antibody**	**Origin**	**Supplier**
TGFβ	mouse	Santa Cruz
TIMP1	mouse	abcam
TIMP2	mouse	abcam
Tissue factor	rabbit	Santa Cruz
TNFα	hamster	Becton Dickinson
Tspan8	mouse	clone D6.1 (1)[a]
Twist	rabbit	Santa Cruz
uPA	rabbit	Santa Cruz
uPAR	rabbit	American Diagnostics
VEGF	rabbit	Santa Cruz
VEGFR1	rabbit	Biotrend
VEGFR2	rabbit	Biotrend
VEGFR3	rabbit	Santa Cruz
vimentin	mouse	Becton Dickinson
vitronectin	mouse	Santa Cruz
Wnt-1	rabbit	Santa Cruz
ZEB1	rabbit	Santa Cruz
HRP-, biotin- or dye-labeled secondary antibodies		Dianova

Table S1C **Chemicals**

**Reagent**	**Concentration**	**Supplier**
CFSE	2.5µM	Invitrogen,Karlsruhe
Cisplatin	in vitro: 1-40µg/ml, in vivo 1µg/ml	Sigma, Munich
collagen I	adhesion: 10µg/ml, invasion: 1mg/ml	Sigma, Munich
collagen IV	adhesion: 10µg/ml	Sigma, Munich
fibronectin	adhesion: 2µg/ml	Sigma, Munich
laminin111	adhesion: 1µg/ml	Sigma, Munich
laminin332	adhesion: 10µg/ml	804G supernatant (3)[a]
matrigel	invasion 1:5	BD, Heidelberg
Phalloidin	0.5µg/ml	BD, Heidelberg
PI	variable	BD, Heidelberg
PMA	10nM, 1h	Sigma, Munich
MMP2 inhib.II	10µg/ml	Calbiochem
MMP9/MMP13 inhib.	10µg/ml	Calbiochem
SP-Dio18(3)	1µM	Invitrogen,Karlsruhe
TACE inhib. (TAPI-2)	10µg/ml	Calbiochem

[a] References

1. Matzku S, Wenzel A, Liu S, Zöller M. Antigenic differences between metastatic and non-metastatic rat tumor variants characterized by monoclonal antibodies. Cancer Res. 1989; 49: 1294-1299.
2. Rana S, Claas C, Kretz CC, Nazarenko I, Zöller M. Activation-induced internalization differs for the tetraspanins CD9 and Tspan8: Impact on tumor cell motility. Int J Biochem Cell Biol. 2011; 43: 106-119.
3. Homma Y, Ozono S, Numata I, Seidenfeld J, Oyasu R. alpha-Difluoromethylornithine inhibits cell growth stimulated by a tumor-promoting rat urinary fraction. Carcinogenesis. 1985; 6: 159-161.

[b] Abbreviations: BD: Becton Dickinson, EAACC: European Association of Animal Cell Cultures, Porton Down, UK, DSHB: Developmental Studies Hybridoma Bank, Iowa, USA

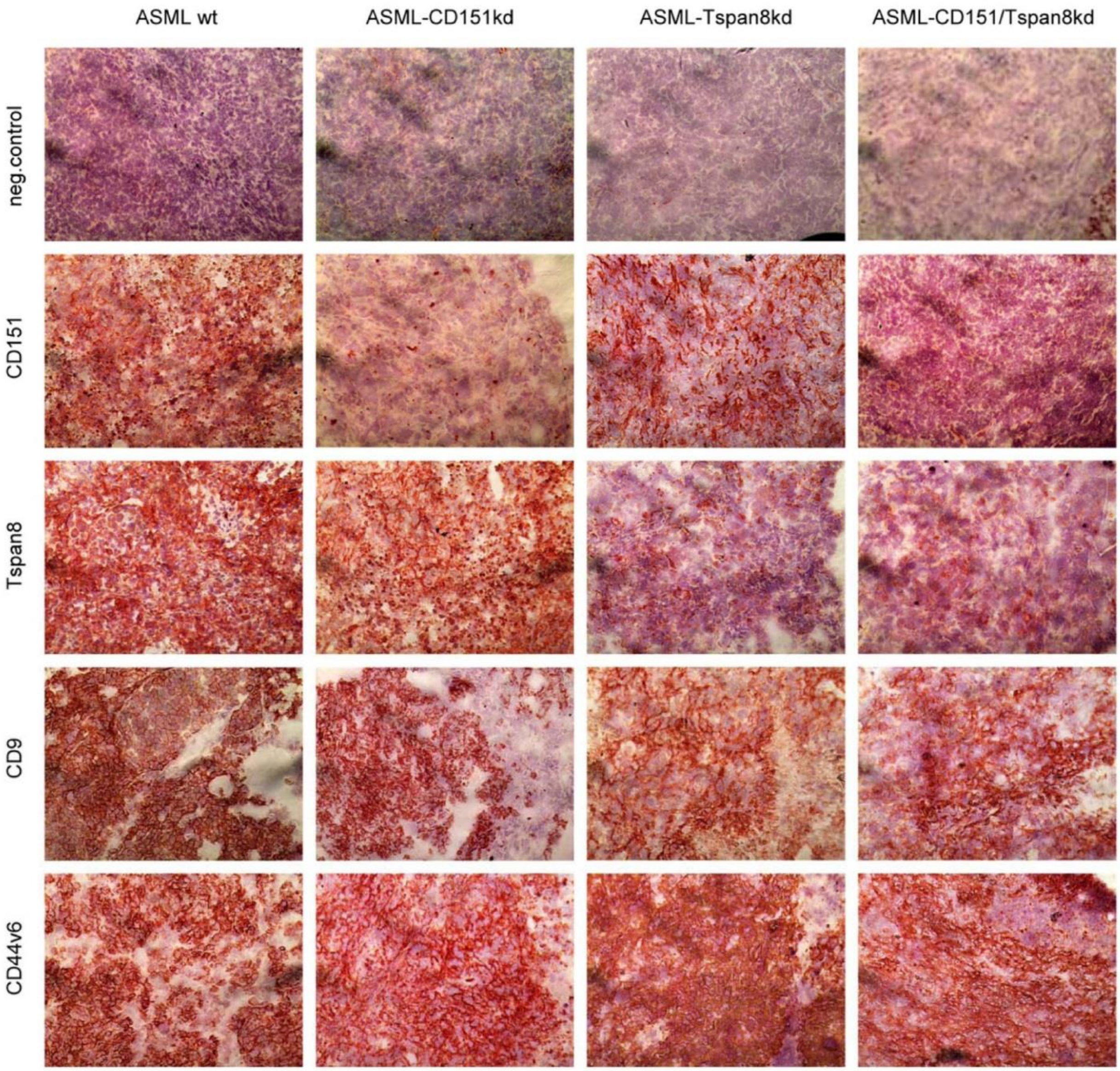

Figure S1: Recovery of ASML cells in draining lymph nodes: Cryosections of the popliteal LN of BDX rats receiving an ifp injection of $1x10^6$ $ASML^{wt}$ or $-CD151^{kd}$ and/or $-Tspan8^{kd}$ cells at autopsy. From the rats receiving ASML-CD151/$Tspan8^{kd}$ cells, the LN of the one rat developing metastasis was selected. Sections were stained for CD151, Tspan8, CD9 and the ASML marker CD44v6. Staining with anti-CD44v6 confirms the stability of the $CD151^{kd}$ and/or $Tspan8^{kd}$ *in vivo*.

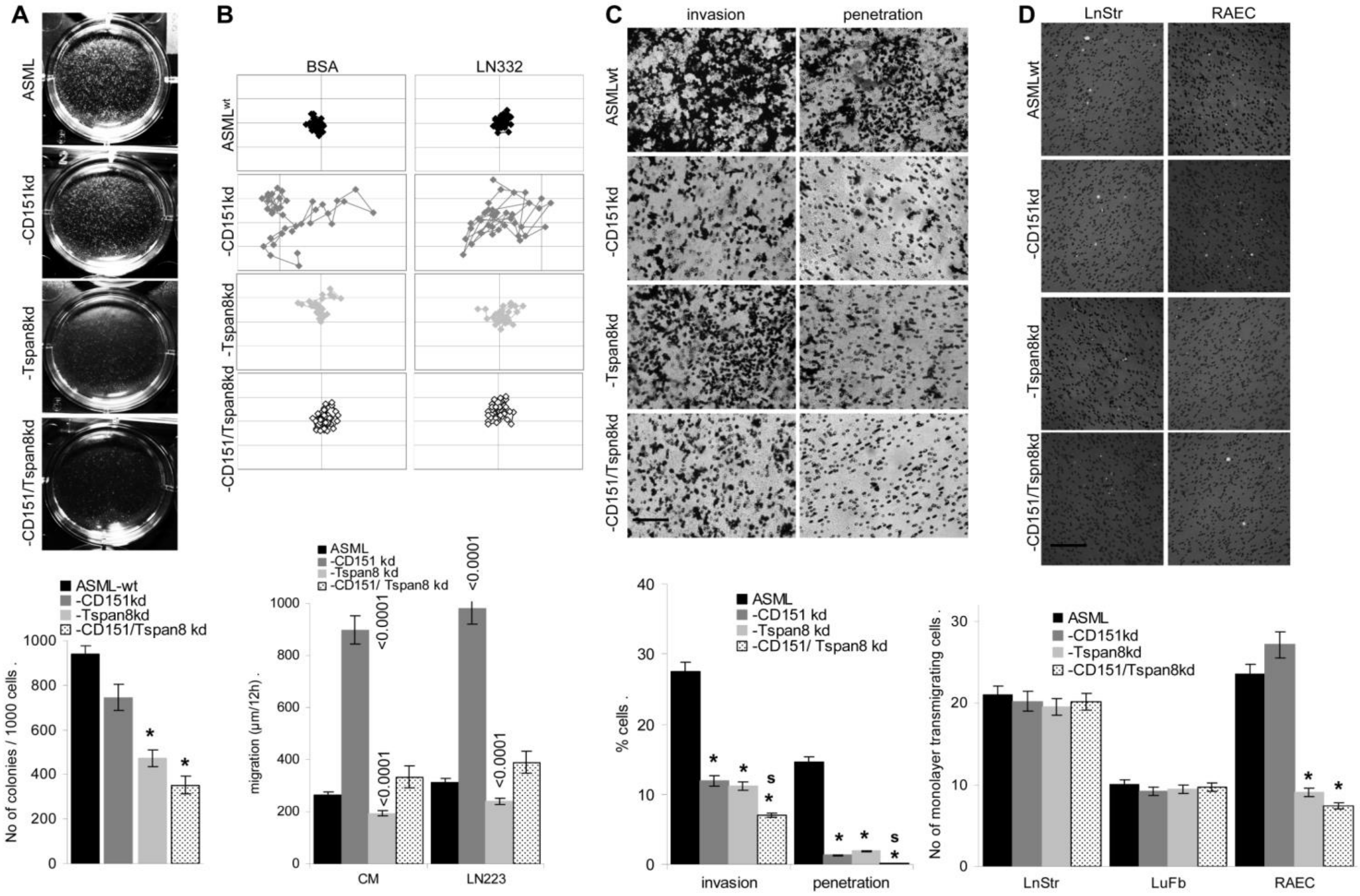

Figure S2: The impact of cellular CD151 and Tspan8 on ASML CIC features, motility and invasiveness: (A) Cloning efficacy in soft agar; representative example and mean number of colonies (mean±SD, 3 assays); significant differences to ASML^{wt} cells: *; (B) Videomicroscopy of ASML^{wt}, - CD151^{kd} and/or -Tspan8^{kd} cell migration on BSA- and LN332-coated plates; representative examples and the mean migration length±SD (20 cells); significant differences to ASML^{wt} cells are indicated; (C) ASML^{wt}, -CD151^{kd} and/or -Tspan8^{kd} cells were seeded on matrigel-coated transwell plate inserts. The mean percent±SD (triplicates) of cells invading the matrigel and penetrating the matrigel and representative examples (scale bar: 150µm); significant differences to ASML^{wt} cells: *, significant differences between ASML-CD151/Tspan8^{kd} versus -CD151^{kd} or -Tspan8^{kd} cells: **s**; (D) CFSE-labeled ASML^{wt}, -CD151^{kd} and/or -Tspan8^{kd} cells were seeded on a monolayer of LnStr, LuFb and RAEC cells in transwell plate inserts. The mean percent±SD (triplicates) of transmigrating ASML^{wt}, - CD151^{kd} and/or -Tspan8^{kd} cells and representative examples (scale bar: 200µm); significant differences to ASML^{wt} cells: *.

In most instances, the loss of function by either the CD151^{kd} or the Tspan8^{kd} becomes dominating in ASML-CD151/Tspan8^{kd} cells: cloning efficacy is impaired; CD151 promoted inhibition of cell motility is also seen in ASML-CD151/Tspan8^{kd} cells; CD151 and Tspan8 equally contribute to matrigel invasion and penetration, the latter being completely abolished in ASML-CD151/Tspan8^{kd} cells; transmigration through an endothelial cell monolayer is severely impaired in ASML-Tspan8^{kd} and -CD151/Tspan8^{kd} cells.

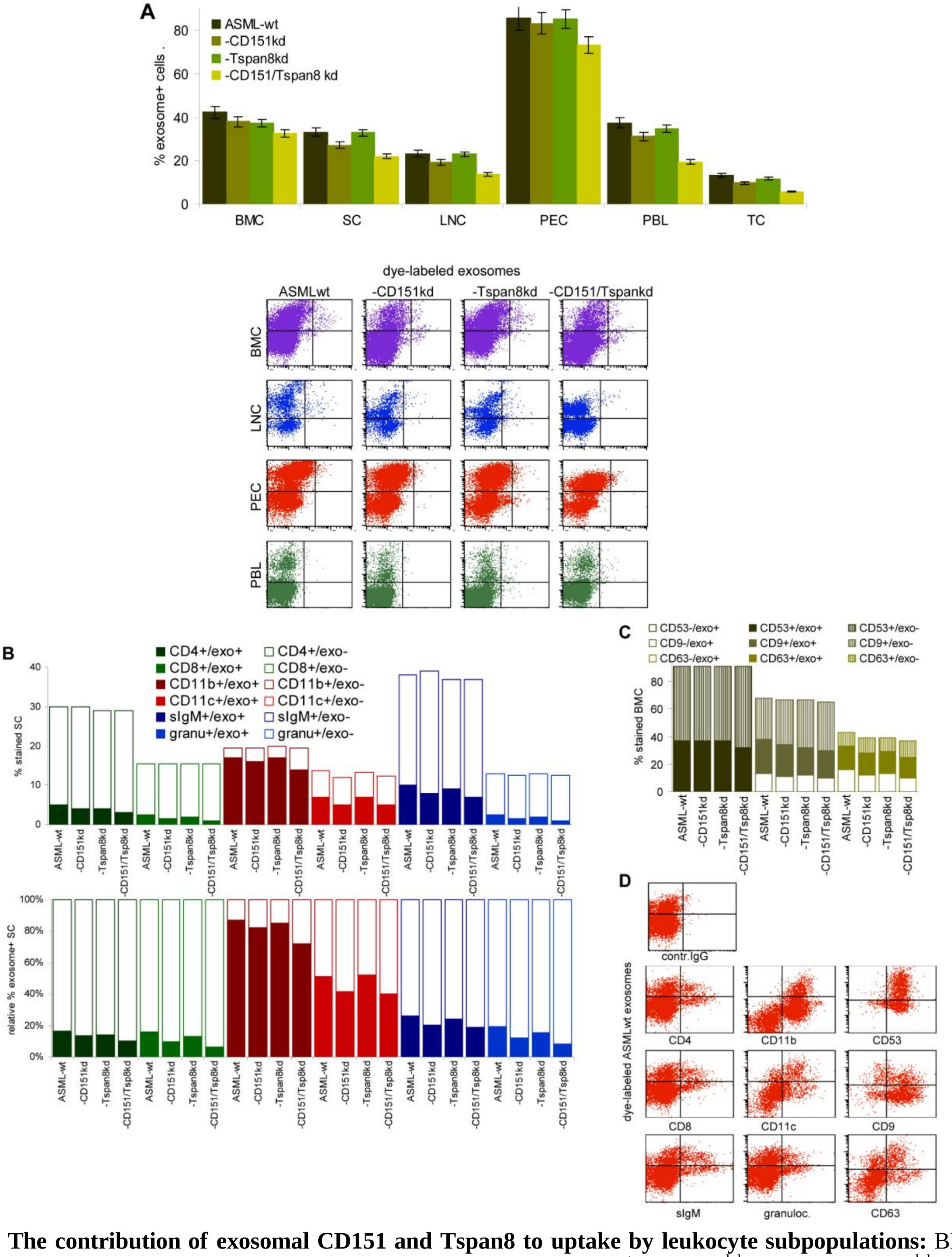

Figure S3: The contribution of exosomal CD151 and Tspan8 to uptake by leukocyte subpopulations: BMC, SC, LNC, PEC and PBL were cocultured for 24h with dye-labeled ASML^{wt}, -CD151^{kd} and/or - Tspan8^{kd} exosomes (25µg/ml). (A) The percent of exosome positive leukocytes (mean±SD, triplicates) and examples; (B) SC were counterstained with leukocyte marker-specific antibodies; the % exosome^{+} subpopulations and the relative distribution of exosome^{+} leukocyte subpopulations are shown; (C) BMC were counterstained with tetraspanin-specific antibodies; the % exosome^{+}/tetraspanin^{+}, exosome^{+}/tetraspanin^{-} and exosome^{-}/tetraspanin^{+} BMC are shown; (C) representative examples.

Tspan8 and more pronounced CD151 contribute to exosome uptake, which is strongly reduced in ASML-CD151/Tspan8^{kd} exosomes. Exosomes are most readily taken up by Mφ and DC, followed by B cells and granulocytes and least efficiently by T cells. The efficacy of the uptake by leukocyte subpopulations is independent from exosomal CD151 and Tspan8, but target cell CD53 appears to contribute to exosome uptake.

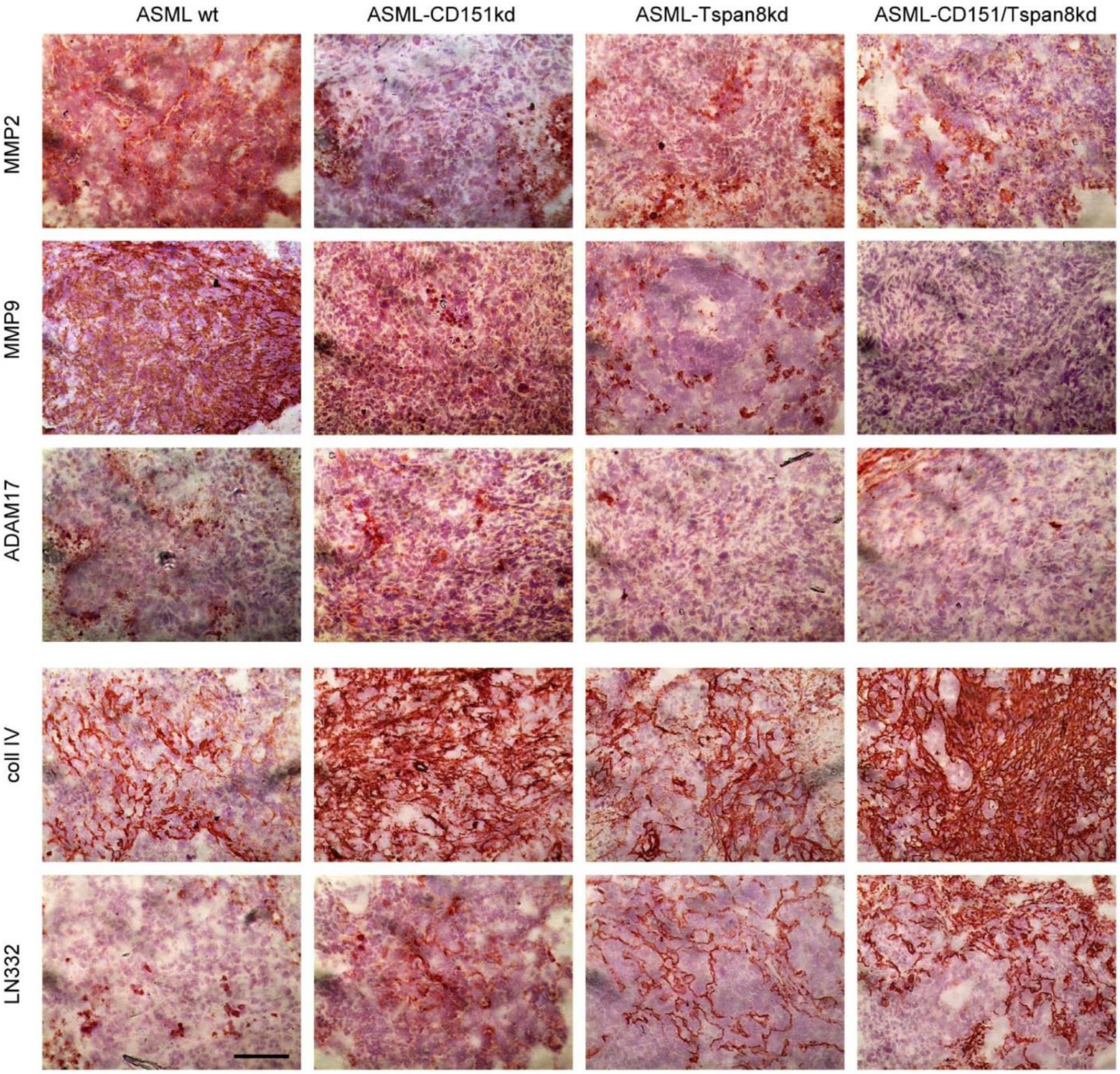

Figure S4: The contribution of exosomal CD151 and Tspan8 to premetastatic niche preparation: Cryosections of the popliteal LN of ASML^{wt}, -CD151^{kd} and/or -Tspan8^{kd} tumor-bearing rats were stained with the anti-MMP2, -MMP9, -ADAM17, -coll IV and -LN332; scale bar: 250µm; representative examples are shown.

Particularly *in vivo*, repeated ASML^{wt} exosome application is accompanied by MMP2, MMP9 and ADAM17 enrichment as well as coll IV and LN332 degradation. Enrichment of MMP2 depends on CD151, enrichment of MMP9 and TACE on Tspan8. Coll IV degradation is completely inhibited in the absence of CD151, LN332-degradation is impaired in ASML-CD151^{kd} and ASML-Tspan8^{kd} exosome-treated rats.

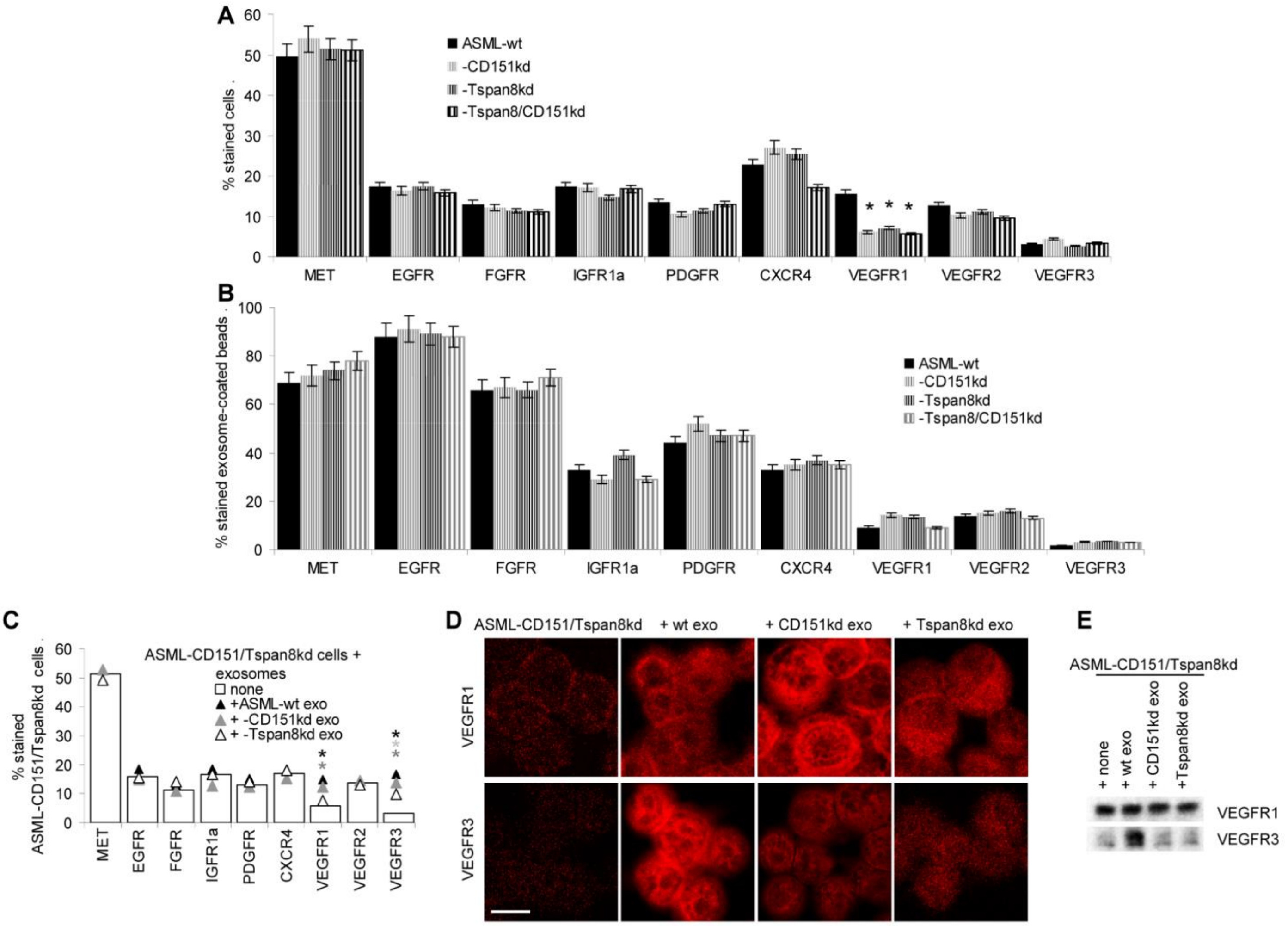

Figure S5: The impact of exosomal CD151 and Tspan8 on RTK expression in ASML-CD151/Tspan8^{kd} cells: (A,B) Flow cytometry of ASML^{wt}, -CD151^{kd} and/or -Tspan8^{kd} cells and exosomes, stained with anti-RTK; mean percent±SD (triplicates) of stained cells / exosome-coated latex beads; significant differences between ASML^{wt} versus -CD151^{kd} and/or –Tspan8^{kd} cells / exosomes: *. (C-E) RTK expression in ASML-CD151/Tspan8^{kd} cells after coculture with exosomes: (C) flow cytometry (mean percent stained cells, 3 assays); significant differences to ASML-CD151/Tspan8^{kd} cells cultured in the absence of exosomes: *, (D) confocal microscopy (scale bar: 10µm) and (E) WB.

With the exception of VEGFR1, expression of RTK is not affected by the CD151^{kd} and/or the Tspan8^{kd} in ASML cells, expression in exosomes is unaltered. Instead, exosomal CD151- and Tspan8- competent exosomes promote VEGFR3 expression in ASML-CD151/Tspan8^{kd} cells.
